# Smartphone App–Based Survey Deployment Patterns and Longitudinal Response Rate: Randomized Controlled Trial

**DOI:** 10.2196/73972

**Published:** 2025-10-10

**Authors:** Yuankai Zhang, Jian Rong, Xuzhi Wang, Eric Schramm, Chathurangi H Pathiravasan, Belinda Borrelli, Jamie M Faro, Emelia J Benjamin, Ludovic Trinquart, Chunyu Liu, Joanne M Murabito

**Affiliations:** 1 Department of Biostatistics School of Public Health Boston University Boston, MA United States; 2 Department of Neurology Chobanian & Avedisian School of Medicine Boston University Boston, MA United States; 3 Care Evolution Ann Arbor, MI United States; 4 Center for Behavioral Science Research Henry M. Goldman School of Dental Medicine Boston University Boston, MA United States; 5 Department of Population and Quantitative Health Sciences UMass Chan Medical School Worcester, MA United States; 6 Boston University’s and National Heart, Lung, and Blood Institute’s Framingham Heart Study Framingham, MA United States; 7 Section of Cardiovascular Medicine, Department of Medicine Chobanian & Avedisian School of Medicine Boston University Boston, MA United States; 8 Department of Epidemiology School of Public Health Boston University Boston, MA United States; 9 Tufts Clinical and Translational Science Institute Tufts University Boston, MA United States; 10 Institute for Clinical Research and Health Policy Studies Tufts Medical Center Boston, MA United States; 11 Section of General Internal Medicine, Department of Medicine Chobanian & Avedisian School of Medicine Boston University Boston, MA United States

**Keywords:** mobile app–based survey, randomized controlled trial, digital device use, mobile health, survey deployment, intervention, longitudinal engagement, mobile phone

## Abstract

**Background:**

Survey fatigue is a common challenge in longitudinal studies, particularly when using smartphone apps to collect survey data. Evidence-based strategies are needed to maintain longitudinal response rates.

**Objective:**

This study aims to evaluate the effect of a more frequent smartphone-administered survey deployment strategy with smaller survey batches on participant response rates over an extended period.

**Methods:**

We conducted a randomized controlled trial (NCT04752657) embedded in the electronic Framingham Heart Study cohorts between June 2021 and December 2023. Participants were randomly allocated to receive a full set of surveys every 4 weeks (control group) or half of the survey set biweekly, such that the full set is completed every 4 weeks (experimental group). Randomization was stratified by age (≤75 y vs >75 y) and phone type (Android vs iPhone). Married couples were assigned to the same group using a blocked randomization approach. The primary outcome was the proportion of surveys returned per participant assessed longitudinally across four periods (baseline to wk 8, wk 8-16, wk 16-24, and wk 24-32), with 19, 17, 16, and 15 unique surveys deployed, respectively. We used mixed-effects regression models with random intercepts to compare the repeated outcome between groups. Stratified analyses by age and sex were performed.

**Results:**

Among 492 participants (mean age 74, SD 6.3 y; 58%, n=284 women, 84%, n=413 non-Hispanic White), there was evidence that the experimental group had higher response rates over time compared to the control group (*P*=.003 for interaction between deployment pattern and time). Both groups showed similar proportions of surveys returned during the first period (75% vs 76%). The experimental group had higher response rates than the control group in subsequent periods (70% vs 67% in wk 8-16, 64% vs 59% in wk 16-24, and 58% vs 50% in wk 24-32). The proportion of participants not returning any surveys increased from 3% to 38% in the control group compared to 1% to 28% in the experimental group across the four time periods. Stratified analyses revealed that among younger participants (≤75 y), the experimental group showed 12% higher survey response rates compared to the control group in the final period, while the difference was minimal among older participants (>75 years). The effect of the deployment pattern was similar for men and women. Three-way interaction analyses revealed no significant differences in the deployment pattern effect over time by age group (*P*=.95) or sex (*P*=.65).

**Conclusions:**

Administering half of the surveys every 2 weeks, as compared to all surveys every 4 weeks, was associated with higher maintained longitudinal survey response rates. This strategy may help mitigate survey fatigue and improve data quality in digital health studies.

**Trial Registration:**

ClinicalTrials.gov NCT04752657; https://clinicaltrials.gov/study/NCT04752657

## Introduction

Advancements in smartphone technology have revolutionized data collection methodologies in health research, offering new opportunities for frequent and real-time assessments of participants’ health status [1]. Smartphone-administered surveys have emerged as powerful tools for collecting self-reported data on various health outcomes, behaviors, and clinical experiences [2]. Digital surveys administered through mobile devices or web platforms enable researchers to gather information more frequently and in real time, potentially reducing recall bias and offering a more convenient and cost-effective approach to capturing participants’ health trajectories [3]. While digital technologies empower personal monitoring of health, promote disease self-management, and maintain patient-provider communication, older adults are often unable to fully access these important benefits [4-6].

A key challenge in longitudinal studies using smartphone apps is maintaining engagement over extended periods, which is crucial for ensuring data quality and study validity [7]. A systematic review and meta-analysis revealed high dropout rates in mobile health interventions, with an average dropout rate of 43% across various study durations [8]. Survey fatigue, in which participants become overwhelmed by too many surveys or repetitive survey questions, can contribute to decreased response rates and potentially compromise data quality [9,10]. These challenges are even more severe among older adults and individuals with chronic health conditions, who may face greater barriers to using digital health technology [11-13]. For instance, older adults may experience age-related declines in cognitive processing speed and vision, making it more difficult to complete app-based surveys consistently [14,15]. Additionally, those with chronic illnesses often deal with symptoms and treatment burden, which can further reduce their capacity or motivation to engage with study tasks [16,17].

Engagement in digital health is multifaceted and can be assessed through a range of metrics, where response rate serves as an important indicator [18-20]. The timing and frequency of longitudinal survey deployment may directly impact participants’ response rate [21]. The optimal timing of survey requests is unclear, as too frequent requests may feel burdensome, while long gaps between scheduled surveys or light touches may lead to participant loss [21-23]. In addition, more frequent deployments enable smaller survey batches, which may reduce participant burden [24]. Previous research has examined various strategies to improve survey response rates in digital health studies, including optimizing survey length, personalized reminders, and incentive structures [25,26]. Some studies have consistently shown that shorter surveys achieve higher response rates [24,27]. Trinquart et al [26] found that personalized notifications are beneficial for device data transmission but not survey completion. However, limited evidence exists on the impact of survey deployment patterns (frequency and number of surveys) on participants’ longitudinal survey response, especially among older adults, who are often underrepresented in digital health research [28]. Understanding the influence of deployment patterns is crucial for designing effective longitudinal digital health research protocols that can sustain participants’ response rate over time.

To address this knowledge gap, we conducted a randomized controlled trial (RCT) to evaluate whether distributing smartphone-administered surveys into smaller, more frequent batches would improve response rates among older adults. Participants were randomized to receive half of the surveys every 2 weeks (experimental group) or all surveys every 4 weeks (control group) across four time periods: baseline to week 8, weeks 8-16, weeks 16-24, and weeks 24-32.

## Methods

### Trial Design

We conducted a 2-arm RCT embedded in the electronic Framingham Heart Study (eFHS) to evaluate the effect of a more frequent survey deployment approach with smaller survey batches on response rates. The trial was registered at ClinicalTrials.gov (NCT04752657). The CONSORT-EHEALTH (Consolidated Standards of Reporting Trials of Electronic and Mobile Health Applications and Online Telehealth) checklist is provided in Multimedia Appendix 1.

### Ethical Considerations

This study was reviewed and approved by the Boston University Medical Campus Institutional Review Board (H-36586, H-40737, and H-32132). All participants provided electronic informed consent for their participation in the eFHS and electronic informed consent for the RCT within the eFHS app. Data were deidentified for analysis. Participants were not compensated for participating in the study.

### Study Setting and Eligibility Criteria

The eFHS is an e-cohort designed to integrate surveys and commercial wearables to capture health data [29]. The eFHS is embedded in the FHS, which is a long-term, community-based cohort study that began recruiting the original cohort of participants in 1948 in Framingham, Massachusetts, to investigate factors and determinants of cardiovascular disease [30-32]. FHS recruited the children of the original participants and their spouses (Offspring cohort; n=5124) from 1971 to 1975 and a cohort of multiple ancestries (Omni 1; n=506) from 1994 to 1998. The Offspring cohort and Omni cohort have undergone examinations every 4 to 8 years, with demographic and clinical data collected at each health examination [33]. At the time of examination 10 (Offspring) and examination 5 (Omni) from 2021 to 2023, participants were invited to the eFHS during the in-person examination at the FHS Research Center in Framingham, or enrolled remotely from home [29]. Eligibility criteria for eFHS included: English-speaking FHS participants who resided in the United States and owned either a smartphone (iPhone with at least iOS 10 or Android phone with at least version 7) or a computer available at home, permission for study notifications and data sharing with the research center, and signed consent. Participants with iPhones were offered a smartwatch to record step count and heart rate data for 1 year following enrollment, while those who already owned a smartwatch were allowed to use their own watch. Participants who enrolled in eFHS were invited to participate in an RCT to test whether delivering half of the surveys every 2 weeks versus all surveys every 4 weeks, administered through smartphones, would improve response rates. Trial participants signed and dated a second consent form. While participants who used either smartphones or computers were eligible for eFHS enrollment, this trial specifically required smartphone ownership: 9 eFHS participants with only computer access were excluded.

The study research technician assisted participants with downloading the eFHS app from the App Store or the Android Google Play Store. Participants received a request for permission to enable notifications and share data with the research center, along with step-by-step instructions for enrollment through the eFHS app. After completing the enrollment and randomization process, participants received their initial smartphone app surveys.

Among 620 eFHS participants, 492 participants were enrolled in the current RCT between June 21, 2021, and June 28, 2023. We conducted the trial over 26 weeks and completed the follow-up of all enrolled participants by December 28, 2023, for both primary and secondary outcomes. Longitudinal surveys were sent out from enrollment (week 0) through week 24 for the control group and through week 26 for the experimental group. Participants in the control group were allowed until week 32 to complete and return their responses to the last batch of surveys, while those in the experimental group were allowed until week 34. Baseline characteristics of participants were obtained using standard protocols and definitions as part of the routine research examination.

Periodic notifications were sent through the eFHS app to notify participants when new surveys became available, to remind participants that surveys were due, and to send a personalized “thank you” greeting when all surveys were completed. Based on our previous work, we sent personalized notifications that included the name of the participant, adaptive messaging based on survey completion status, and the eFHS study technician’s name to improve survey response rate [26]. We selected the notification message randomly from a message bank (Multimedia Appendix 2). For example, we sent a personalized notification when a survey was due in the next 7 days, such as “Mr. (S) [FHS participant last name], we missed your survey responses last week. We would really appreciate it if you could complete your surveys.” If all surveys were completed, participants received a thank you message selected at random, such as “Mr. (S) [FHS participant last name], way to go! We received your surveys. Thank you!” To ensure that message variation did not confound our results, both experimental and control groups received the same types of messages selected from the same message bank, with random message selection occurring independently of group assignment. Since the experimental group received survey batches more frequently, they consequently received notification messages more often as well.

Additionally, 1 week after randomization, we contacted all participants by telephone to inquire about any technical issues with the eFHS app. Throughout the study, support staff provided assistance to participants. When interacting with participants to address technical questions, the support staff adhered to standardized scripts to ensure consistency and minimize potential bias.

### Surveys and Randomized Deployment Approaches

The MyDataHelps Designer platform (CareEvolution) was used to create the eFHS smartphone app surveys within the MyDataHelps mobile app container. The MyDataHelps app hosts a variety of surveys designed to be sent out on enrollment and at various intervals after enrollment to participants to answer health surveys administered through the app. A previous study in the eFHS reported that 469 participants who returned the Mobile App Rating Scale survey rated app functionality and aesthetics highly (total Mobile App Rating Scale score=8.6 on a 1-10 scale), and those who completed the System Usability Scale survey indicated high satisfaction with the app’s design and ease of use [29]. In addition, the app included the participant’s account, where the signed consent form can be viewed, and a dashboard to provide participants with a thank you message and survey completion status.

Surveys were chosen for relevance to the health of older adults (Multimedia Appendix 3). The short form of the Patient-Reported Outcomes Measurement Information System [34] allows self-reported assessments of anxiety, depression [34], fatigue [35], sleep quality [36], physical function, pain, cognitive abilities, and cognitive function [37]. Most surveys typically consisted of 4-item multiple-choice questions with responses ranging from “never” to “always” or “not at all” to “very much.” Additionally, a modified version of the Michigan Body Pain Map [38] was used to collect information on chronic pain through a pictorial representation of the human body. We also included surveys of physical activity level [39], mobility outside the home [40], and occurrences of falls and hospitalizations.

Task-based surveys included smartphone-operated cognitive and physical assessments: a Trail Making Test requiring participants to tap a series of alternating numbers and letters [41,42], a Victoria Stroop Test had four subtests that required participants to select the color of the matching color block, select the color of the word printed in black font, and select the color of words printed in incongruent colors, and select the word printed in incongruent colors unless the word was underlined [43], a gait task measuring walking time [44], and a two-finger tapping task assessing tapping speed and accuracy [45]. Due to the unavailability of these task-based surveys on Android phones, Android users contributed data only to the other question-based surveys (Multimedia Appendix 4).

**Figure 1:**
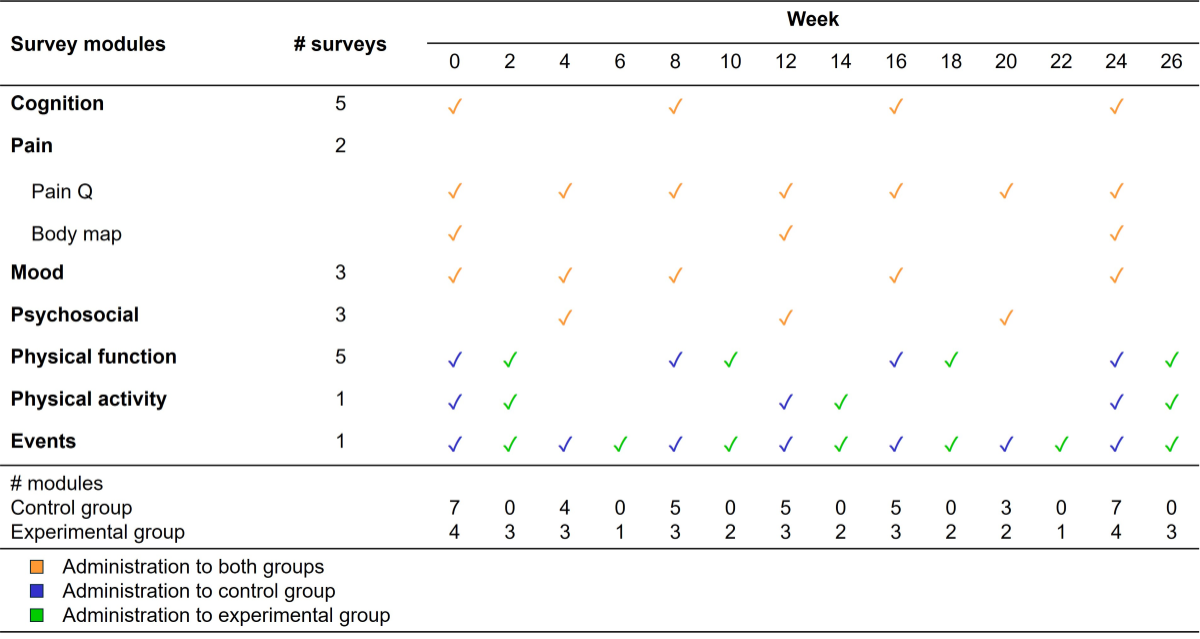
Timeline and pattern of survey module administration in the experimental (half batch every 2 wk) and control (full batch every 4 wk) groups. Both groups received the same number of surveys within each of the 4 time periods. The following smartphone-administered survey tasks were only available on the iPhone: trail making, Stroop, two-finger tapping in the cognition module, and gait in the physical function module. Surveys were administered from week 0 through week 24 for the control group and through week 26 for the experimental group, as shown. Participants in the control group were allowed until week 32 to complete and return their responses to the last batch of surveys, while those in the experimental group were allowed until week 34.

The surveys were scheduled to be sent out at enrollment (baseline, week 0) and at various intervals afterward through week 26 (Figure 1 and Multimedia Appendix 4). Participants were randomized to receive surveys as per the two different deployment patterns to compare response rates. At each scheduled time point, participants received a batch containing multiple surveys of different types. The control group received all surveys every 4 weeks, while the experimental group received half of the surveys every 2 weeks. Both groups received the same number of surveys within each time period. The deployment pattern was consistent across both groups for the cognition, pain, mood, and psychosocial modules. However, the deployment of the physical function, physical activity, and events surveys was shifted for the experimental group as compared to the control group, resulting in smaller but more frequent survey batches. Participants were allowed to complete a survey batch from its deployment until the next wave, with time intervals varying across modules based on their deployment schedule. For the trial, we followed participants through week 32 for the control group and week 34 for the experimental group to allow for survey responses to the last batch of surveys.

### Outcomes

We prespecified 4 survey periods. Both groups received the same number of surveys within each time period (Figure 1 and Multimedia Appendix 4). Period 1 encompassed all survey modules deployed from baseline up to week 8 (22 surveys per iPhone users; 18 surveys per Android users), period 2 from week 8 up to week 16 (19 surveys per iPhone users; 15 surveys per Android users), period 3 from week 16 up to week 24 (18 surveys per iPhone users; 14 surveys per Android users), and period 4 from week 24 to week 32 (15 surveys per iPhone users; 11 surveys per Android users).

We quantified survey response rates for two outcomes. The primary outcome of the participant’s survey response rate was calculated as the proportion of surveys returned per participant within each time period, including both partially and fully completed surveys. For question-based surveys with 4 or more questions, a completion rate of at least 75% was considered partially completed (Multimedia Appendix 4). For the pain survey with 3 questions and the event survey with 2 questions, we used 66% and 50% as thresholds, respectively (Multimedia Appendix 4). The secondary outcome was the proportion of questions or tasks completed per participant in each time period, calculated as the number of completed questions or tasks divided by the total number of questions or tasks across all surveys combined. While the primary outcome captures overall survey participation (whether surveys were returned), the secondary outcome provides a more granular measure of participant engagement by quantifying the completeness of responses within returned surveys. Participants may return surveys but leave some questions unanswered due to survey fatigue, difficulty with specific questions, or technical issues. The secondary outcome thus helps differentiate between participation and the thoroughness of survey completion. We conducted a longitudinal analysis to compare the mean proportions of the primary and secondary outcomes across the 4 time periods.

### Random Allocation

Participants in the current trial were randomly allocated to one of the two groups using randomization lists generated by a statistician with randomly permuted blocks of varying sizes. The FHS Offspring cohort includes spouses. Therefore, for married couples participating in eFHS and enrolled in the trial, a simple blocked randomization approach was used to ensure that the spouses were always assigned to the same group, as it was assumed that they would be likely to complete the surveys together. For other participants, randomization was stratified according to participants’ age (≤75 y vs >75 y) and type of phone (Android vs iPhone). The stratification approach was informed by a previous study using the FHS Generation 3 cohort, which found that both age and phone type were strongly associated with survey return and that age modified the effect of time on survey completion [46]. While the couple randomization was not stratified by age or phone type, it was expected that the random allocation would still achieve a balance of these factors. The randomization process was implemented centrally through the eFHS app. eFHS researchers and statisticians were blinded to the group assignment.

### Sample Size

We calculated the sample size needed to detect differences in survey response rates between randomization groups over time. We assumed a correlation of the repeated measures of 0.6 and an SD on the proportion of surveys returned of 20%. A sample size of 240 participants per group would provide 83% power to detect a between-group difference in slopes of at least 2% for the proportion of returned surveys over time, using a 2-sided test with an α of 0.05. The study would have a limited power of 31% to detect a three-way interaction effect size of 2% between subgroups across 4 time points with 480 participants in total.

### Collection of Demographic Data

Participants’ demographic data were obtained from comprehensive data collection during routine FHS research examinations. The demographic variables analyzed in this study included age, sex, race or ethnicity, education level, marital status, employment status (retired vs not retired), annual household income, and self-rated health status. They were collected at examination 10 for the Offspring cohort and examination 5 for the Omni 1 cohort. Phone type (iPhone vs Android) and smartwatch use were documented during eFHS enrollment.

### Statistical Methods

Baseline characteristics of participants were presented as mean (SD) for continuous variables or n (%) for categorical variables. We fitted a mixed-effects regression model with random intercepts to compare the mean primary and secondary outcomes between randomization groups across time periods. Analyses were in intention-to-treat so that all participants randomized to one of the groups were analyzed within that group. The model included fixed effects for group assignment (experimental vs control group), time periods, age group (>75 y vs ≤75 y), phone type (iPhone vs Android), and a multiplicative interaction of group by time periods. Random intercepts were included to account for both within-individual correlation over time and intraclass correlation within spouses. An unstructured covariance structure was used to model the correlation between repeated measurements. For the comparisons at any specific time period, we estimated the difference in outcomes between groups using mixed-effects regression. In primary analyses, we used a linear mixed-effects regression model to analyze the outcomes, which were continuous proportions. In sensitivity analyses, we defined a binary outcome by categorizing each survey as returned or not returned, and we modeled this repeated binary outcome by a logistic mixed-effects model.

Furthermore, a priori stratified analyses were performed to assess and compare the primary and secondary outcomes in subgroups defined by age group (>75 y vs ≤75 y) and sex (women vs men). We also applied three-way interaction analyses to examine the effect of survey deployment pattern over time across the subgroups.

All analyses were conducted in R (version 4.3.0; R Core Team). Two-sided tests were performed for all analyses; we considered *P*<.05 as statistically significant.

## Results

### Participant Characteristics

**Figure 2:**
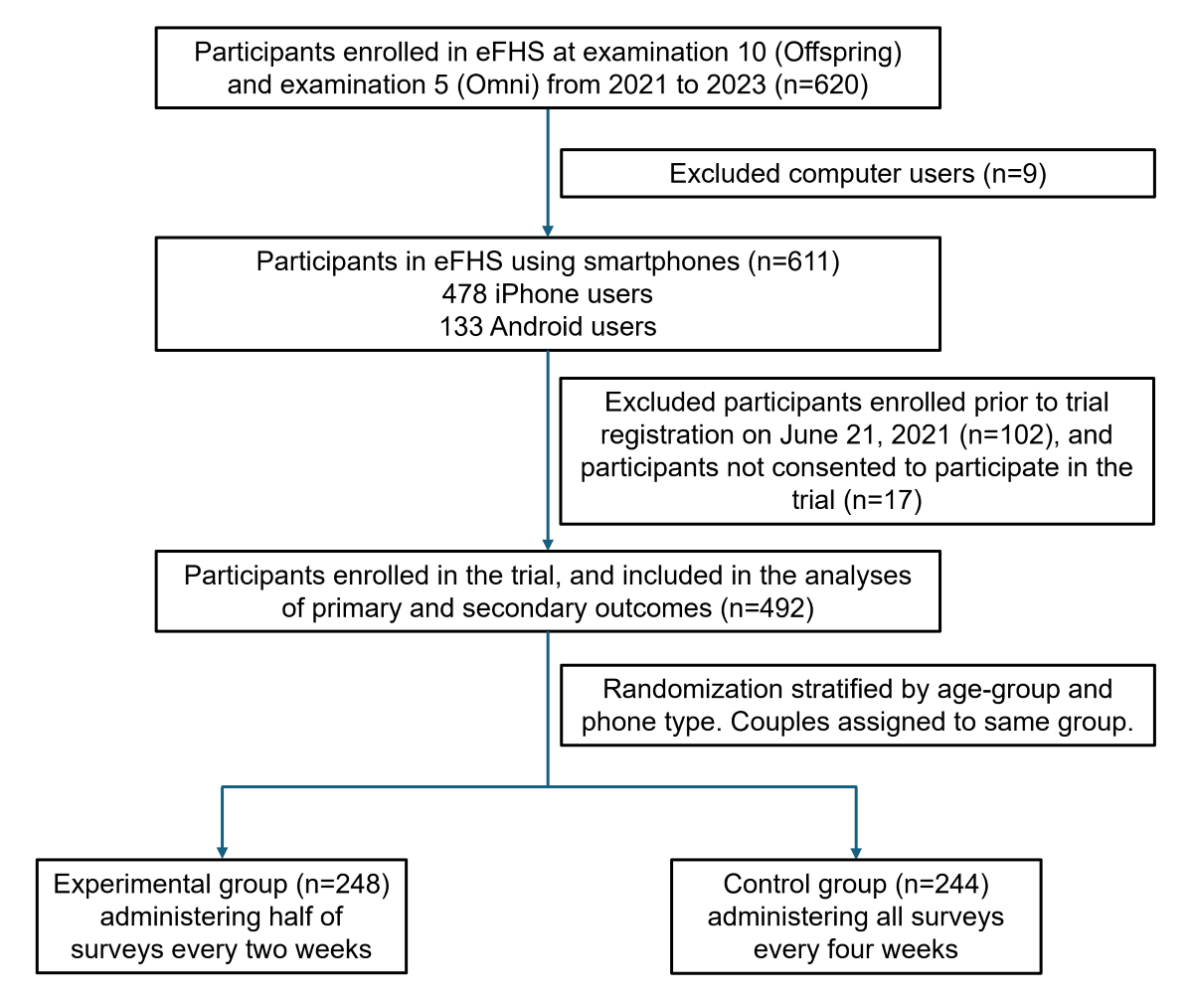
Flow diagram of participant enrollment and randomization in the trial. eFHS: electronic Framingham Heart Study.

A total of 492 participants were enrolled in the trial, with 248 participants in the experimental group and 244 participants in the control group, and 20% (n=98) participating as part of a couple (Figure 2). Table 1 summarizes the characteristics of the participants. The mean age of participants was 74 (SD 6.3; range 55-92) years, 58% (284/492) were women, 84% (413/492) were non-Hispanic White, 67% (327/492) had a bachelor’s degree or higher, and 69% (337/492) considered their health to be very good or excellent. Following randomization, the experimental and control groups had similar baseline characteristics (*P*>.05 for all comparisons). In addition, characteristics of participants in the trial were similar to other eFHS participants (n=128) who chose to receive surveys or smartwatches but were not part of the trial (Table S1 in Multimedia Appendix 5).

**Table 1:** Baseline characteristics of participants enrolled in the trial (n=492).

Characteristics		Overall (n=492)	Experimental group (n=248)	Control group (n=244)	*P* value^a^	
Age (years), mean (SD)		73.9 (6.3)	74.0 (6.2)	73.9 (6.3)	.82	
Women, n (%)		284 (57.7)	148 (59.7)	136 (55.7)	.43	
**Race or ethnicity, n (%)**						.74
	Non-Hispanic White	414 (84.1)	209 (84.3)	205 (84)		
	Black	30 (6.1)	17 (6.9)	13 (5.3)		
	Asian	35 (7.1)	17 (7.9)	18 (7.4)		
	Hispanic	13 (2.6)	5 (2.0)	8 (3.3)		
Bachelor’s degree or higher, n (%)		327 (66.5)	166 (66.9)	161 (66)	.90	
Marital status, married, n (%)		340 (69.4)	162 (65.6)	178 (73.3)	.08	
Participating as a couple, n (%)		98 (19.9)	49 (19.8)	49 (20.1)	≥.99	
Phone type, iPhone, n (%)		382 (77.6)	190 (76.6)	192 (78.7)	.66	
Smartwatch user, n (%)		287 (60.2)	151 (62.9)	136 (57.4)	.25	
Retired, n (%)		337 (68.6)	170 (68.5)	167 (68.7)	≥.99	
**Annual income, n (%)**						.83
	<US $35,000	42 (10.7)	23 (11.7)	19 (9.8)		
	US $35,000-$74,999	110 (28.1)	55 (27.9)	55 (28.4)		
	≥US $75,000	239 (61.1)	119 (60.4)	120 (61.9)		
Subjective health, very good or excellent, n (%)		337 (68.6)	165 (66.5)	172 (70.8)	.36	

^a^*P* compares the experimental and control groups. Continuous variables were compared using the 2-tailed *t* test, and categorical variables were compared using the chi-square test.

### Effect of Survey Deployment Patterns on the Survey Response Rate

We found that the more frequent deployment with fewer surveys per batch (experimental group) had superior response rates over time compared to the less frequent deployment with more surveys per batch (control group) for both primary and secondary outcomes (Table 2; *P*=.003 and *P*=.002 for interaction between deployment pattern and time, respectively). For the primary outcome (proportion of surveys returned), the two groups were comparable from weeks 0-8 (75% vs 76%), then showed progressively larger differences of 3%, 5%, and 8% in weeks 8-16 (70% vs 67%), weeks 16-24 (64% vs 59%), and weeks 24-32 (58% vs 50%). The secondary outcome (proportion of questions or tasks completed) was highly correlated with the primary outcome (Figure S1 in Multimedia Appendix 5; *R*=0.99). Hence, a similar trend was observed for the secondary outcome, with similar response rates during weeks 0-8 (77% vs 78%), followed by increasing differences across weeks 8-16 (71% vs 69%), weeks 16-24 (65% vs 61%), and weeks 24-32 (59% vs 52%). In addition, the proportion of participants not returning any surveys increased from 2% in weeks 0-8 to 33% in weeks 24-32, with a consistently higher dropout rate in the control group (3% to 38%) compared to the experimental group (1% to 28%; Figure S2 in Multimedia Appendix 5). These results indicated that while both groups showed declining survey response rates over time, the experimental group (more frequent but fewer survey deployment patterns) displayed a slower decline over the four periods compared to the control group.

**Table 2:** Longitudinal comparison of survey response rates between experimental (half batch of surveys every 2 weeks) and control (full batch of surveys every 4 weeks) groups across four time periods (n=492).

Time period^a^		Experimental group (n=248)	Control group (n=244)	Difference in proportions^b^	*P* value of group-by-time interaction	
**Primary outcome (%)**^c^**: proportion of surveys returned** (**95% CI**)						.003
	1	75.4 (70.2 to 80.6)	75.6 (70.3 to 80.9)	–0.2 (–7.0 to 6.5)		
	2	69.5 (64.5 to 74.4)	67.2 (62.2 to 72.2)	2.3 (–4.0 to 8.6)		
	3	63.5 (58.6 to 68.5)	58.7 (53.7 to 63.8)	4.8 (–1.5 to 11.1)		
	4	57.6 (52.4 to 62.8)	50.3 (45.0 to 55.6)	7.3 (0.5 to 14.0)		
**Secondary outcome (%)^c^: proportion of questions or tasks completed (95% CI)**						.002
	1	77.0 (71.8 to 82.2)	77.8 (72.5 to 83.1)	–0.8 (–7.6 to 6.0)		
	2	71.1 (66.2 to 76.1)	69.2 (64.2 to 74.3)	1.9 (–4.4 to 8.2)		
	3	65.3 (60.3 to 70.2)	60.7 (55.7 to 65.7)	4.6 (–1.7 to 10.9)		
	4	59.4 (54.2 to 64.6)	52.1 (46.8 to 57.4)	7.3 (0.5 to 14.1)		

^a^Time period: 1=weeks 0-8; 2=weeks 8-16; 3=weeks 16-24; and 4=weeks 24-32.

^b^Differences estimated as outcomes of the experimental group minus outcomes of the control group.

^c^Estimated outcomes were derived from a linear mixed-effects model analyzing repeated measurements of primary or secondary outcomes across four time points per participant. The model included fixed effects for randomization group, timepoint (time period 1-4), age-group (≤75 y vs >75 y), phone type (iPhone vs Android), and a randomization group-by-time interaction. Random intercepts were included for each couple and individual to account for correlation within couples and repeated measures of individuals.

In a sensitivity analysis, we coded the outcome of each survey as a binary variable, that is, returned or not returned. We used these binary outcomes instead of using the proportion in our analyses. Since only approximately 1% of returned surveys were partially completed, we did not analyze partially versus fully completed surveys and categorized both as “returned.” The significant interaction between time and group (Table S2 in Multimedia Appendix 5; *P*<.001) demonstrated that the experimental group had a slower decline in survey response rate over time compared to the control group. The odds ratios (experimental vs control group) between groups increased progressively over time, from 1.00 (95% CI 0.93-1.07) in the first time period to 1.08 (95% CI 1.01-1.15) in the final period (Table S2 in Multimedia Appendix 5), indicating higher odds of survey completion in the experimental group after the first period.

The effects of survey deployment pattern over time were explored across subgroups of biological characteristics (age and sex) through three-way interaction analyses. None of these interactions were significant (*P*=.95 for age groups; *P*=.65 for sex), indicating that the deployment pattern effect over time was not significantly different across participant subgroups by age groups (≤75 y [n=288] vs >75 y [n=204]) and sex (women [n=284] vs men [n=208]). However, with only 31% power to detect a three-way interaction effect, this study was underpowered to formally test whether the intervention effect significantly differed between strata. In the age-stratified analyses, we observed that, among younger participants (≤75 years), the experimental group showed improved survey return rates over time compared to the control group (12% higher in the final period), whereas, among older participants (>75 years), there were minimal differences between groups (Table S3 in Multimedia Appendix 5). In both women and men, the experimental group maintained higher response rates than the control group in later time periods: among women, there was a 6% between-group difference in the final period, whereas among men, there was an 8% difference between groups in the final period (Table S4 in Multimedia Appendix 5).

## Discussion

### Principal Findings

This RCT, embedded within the eFHS Offspring and Omni Study, provides insight into the impact of survey deployment patterns on longitudinal response rate among older adults using smartphone-administered health surveys. Our findings demonstrated that administering half of the surveys every 2 weeks (experimental group) was associated with a maintained response rate compared to administering all surveys every 4 weeks (control group). The findings suggest that the survey deployment patterns can significantly influence participants’ response rate over time, with potential implications for the design of digital health research protocols.

The trial design maintained the total survey burden constant between groups, isolating the effect of deployment pattern from overall workload. Several mechanisms may explain the superior response rates observed in the experimental group. First, the biweekly deployment pattern likely reduced cognitive burden by presenting fewer surveys per session, making each interaction less likely to be overwhelming [9,47,48]. Additionally, the more frequent touchpoints may have helped maintain participant engagement through regular reminders of their study participation, creating a routine that integrated survey completion into participants’ schedules. The shorter time interval between deployments may have also reduced the likelihood of participants losing motivation between survey waves and reinforced the connection between participants and the research through consistent study contact [49,50].

Our results contribute to the growing body of literature on optimizing digital health research methods, particularly for older adult populations. To date, limited studies have investigated the impact of survey deployment patterns on response rates. A systematic review of RCT participation burden found that more frequent questionnaires increased patient workload, where they compared daily versus weekly assessments [51]. However, some prior studies suggested that frequent follow-up combined with other engagement strategies can increase survey response rates [52,53]. These mixed findings highlight the complexity of optimizing deployment frequency: too frequent may increase burden, while too infrequent may lead to loss to follow-up. The optimal deployment frequency likely varies by survey type and length and may require further exploration across different study contexts. This study provides insights into this topic by specifically examining the impact of survey deployment patterns (half batch of surveys every 2 weeks vs full batch of surveys every 4 weeks) on participants’ response rate among older adults (mean age 74, SD 6.3 y). The observed decline in survey response rates over time is consistent with findings from other longitudinal health studies [21,54]. However, our results suggest that more frequent but smaller survey batches may mitigate this decline to some extent, potentially improving adherence and reducing survey fatigue [55]. This approach appears to be less burdensome compared to less frequent, larger survey batches. In addition, these more frequent touches with participants may help maintain connection to the study [22,23].

In this trial, while the experimental group showed less dropout than the control group, both groups experienced increasing dropout rates over time, with cumulative dropout reaching 28% and 38%, respectively, by weeks 24-32. However, these dropout rates are lower than those reported in many other digital health studies. For instance, Meyerowitz-Katz et al [8] analyzed 17 mobile health studies and found an average dropout rate of 43%, where studies varied widely in duration, ranging from 2 weeks to 1 year, with nearly one-third of the studies lasting 1 month or less. Daniore et al [56] reported a median study completion rate of 48% among 19 remote digital health studies lasting 12 weeks or less, highlighting that retention rates as low as 10% are common in digital health research [56]. The persistence of dropout even with our deployment strategy reflects the inherent challenges of maintaining long-term retention in remote digital health research [57].

Older adults do not adopt new and emerging technologies as readily as younger people [58-60]. However, while younger adults often demonstrate higher initial engagement with technology, retention and adherence fall off over time compared to adults aged 60 years and older [61]. Older age has been observed to be associated with improved retention in eight remote digital health studies where those aged 60 years and older were the least represented age-group, with shorter participant retention times (median across 8 studies varied from 2 to 26 days) than investigated in this trial [62]. A similar pattern was also observed in a previous study on middle-aged participants in FHS [46]. Understanding the predictors of older adults’ internet use and digital competence has become increasingly important as people live longer and the age profile of societies rapidly changes [63]. While many previous studies broadly classify “older adults” as those older than 60 years, our study population (mean age 74, SD 6.3 y) provides evidence for a substantially older demographic [61,62]. Old-age diversity is underrepresented in digital health research, and older individuals were more likely to be nonscreened, nonrecruited, or decliners in these studies [28]. This study revealed differences in response rate between older age-groups (higher response rate for those ≤75 y), which deepens our understanding of strategies to maintain survey response rates among older adults in digital health research. National survey data and data from large hospital centers identify older age as a factor associated with declines in wearable device use, consistent with less interest or comfort with technology [64]. These findings underscore the need to apply age-specific approaches in digital health research. When promoting technology adoption among older adults, both the technology’s features and the capabilities of older users should be considered, along with consideration for the incorporation of appropriate technical support [58]. Personalized learning approaches and aids that respect older adults’ pace and connect to their real-life needs may improve their engagement with digital technologies [65].

### Strengths and Limitations

This study benefits from several key strengths. The eFHS is embedded within the long-standing FHS, whose participants have been involved for decades. This long-term engagement has fostered trust between participants and researchers, likely contributing to higher retention rates than typically seen in e-cohorts. For example, among our sample with a mean age of 74 (SD 6.3) years, more than 65% (320/492) of participants continued to return surveys in the last period. Additionally, this study’s 26-week duration provides valuable insights into extended engagement patterns, offering a more comprehensive view of participant behavior compared to shorter-term digital health studies. Furthermore, the nested design of eFHS within FHS allows participants to be well characterized at in-person research center examinations.

Several limitations should be considered when interpreting the results of this study. First, the generalizability of our findings may be limited due to the characteristics of our study cohort. The eFHS participants were predominantly older, non-Hispanic White, English-speaking, well-educated, and resided primarily in the New England region of the United States. Moreover, eFHS participants were generally healthier than the broader FHS cohorts. The response rate patterns observed in this trial may be influenced by the participant characteristics. For example, higher education levels are associated with greater health literacy and technology adoption, potentially facilitating survey completion and reducing barriers to technology access and use [66,67]. The long-standing relationship between FHS participants and researchers likely enhanced trust and commitment, contributing to the relatively high response rates. Therefore, our findings may not be fully applicable to more diverse populations, younger participants, or those with different socioeconomic backgrounds or specific health conditions. Second, while the more frequent survey deployment pattern with smaller batches (administering half of the surveys every 2 weeks) was associated with an increased response rate, it introduced a temporal misalignment in end point assessments. This resulted in some instruments being assessed up to 2 weeks earlier or later compared to others, potentially introducing variability in the timing of data collection. This temporal discrepancy should be considered when interpreting the results and may have implications for studies where precise timing of assessments is critical. Third, our subgroup analyses by age and sex had limited statistical power to detect the three-way interaction and should be viewed as exploratory findings. Additionally, unmeasured factors such as technology literacy may have influenced response rates within subgroups, potentially masking interaction effects. Fourth, this trial focused on longitudinal response rates as main indicators of participant engagement, but other potentially informative engagement metrics, such as retention rates, time to survey completion, or time spent on each survey, were not assessed. While notification messages were randomly selected from the same message bank, we did not examine potential interactions between message type and deployment pattern. Furthermore, this study was limited to smartphone users and may not be generalized to digital health research using other mobile or electronic devices, such as tablets or desktop computers, which may have different use patterns and user interfaces.

### Future Work

Future studies should investigate the impact of survey deployment patterns across diverse populations, including those with varying demographic backgrounds and specific health conditions. Additionally, research is needed to explore the optimal balance between survey frequency and quantity. While this study compared two specific patterns, future investigations could examine a broader spectrum of deployment patterns.

Larger studies with adequate statistical power are needed to confirm our exploratory subgroup findings and should collect data on additional factors, such as technology literacy, to better understand differential effects across participant characteristics. Future research should also incorporate other complementary engagement metrics beyond response rates, including retention rates, time to survey completion, and time spent on surveys, to develop a more comprehensive understanding of how deployment patterns affect various dimensions of participant engagement in digital health research.

Our findings underscore the potential of digital technology to engage older adults in health research. The high response rates, even among older adults, demonstrate the feasibility of remotely collecting important health data on mood, cognition, and physical function from older populations. This is particularly significant as technology increasingly empowers older individuals to self-monitor chronic conditions at home [68]. It also encourages implementation of decentralized RCTs that leverage remote participation and data collection, potentially expanding research access to traditionally underrepresented populations while reducing participant burden [69].

Digital health studies offer a promising avenue for research participation, particularly for those who may struggle with in-person examinations, and they can extend outreach to improve access and representation in health research and clinical trials [70-72]. As digital methods become increasingly prevalent in health research, optimizing strategies for sustained participant engagement is crucial. Identifying effective strategies to maintain high response rates can improve the quality and representativeness of data collected through digital devices, ultimately enhancing the understanding of health trajectories in populations [73].

### Conclusions

In conclusion, this RCT provides evidence that survey deployment patterns influence longitudinal response rate in smartphone-based health research among older adults. The strategy of administering smaller, more frequent survey batches shows promise in maintaining higher response rates over time compared to larger, less frequent batches.

## Data Availability

The datasets generated and analyzed during this study are available from the corresponding author on reasonable request and will be available at public repositories (DbGaP and BioLINCC).

## Appendix

Multimedia Appendix 1 CONSORT-eHEALTH checklist (V 1.6.1).

Multimedia Appendix 2 Message bank of periodic notifications.

Multimedia Appendix 3 Screenshots of surveys.

Multimedia Appendix 4 Survey deployment patterns.

Multimedia Appendix 5 Secondary and sensitivity analyses.

## Abbreviations

CONSORT-EHEALTH: Consolidated Standards of Reporting Trials of Electronic and Mobile Health Applications and Online Telehealth
eFHS: electronic Framingham Heart Study
RCT: randomized controlled trial
