# Supplementary material for: Smartphone App–Based Survey Deployment Patterns and Longitudinal Response Rate: Randomized Controlled Trial
Source: J Med Internet Res. 2025 Oct 10;27:e73972. doi: 10.2196/73972 (PMC12552817; doi:10.2196/73972)
Supplement: Multimedia Appendix 3 [file jmir_v27i1e73972_app3.docx]

**Mood Survey:**

**
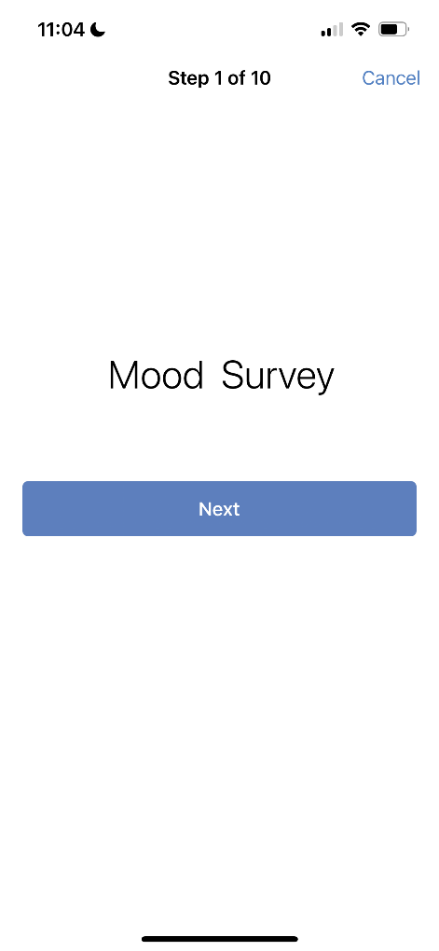

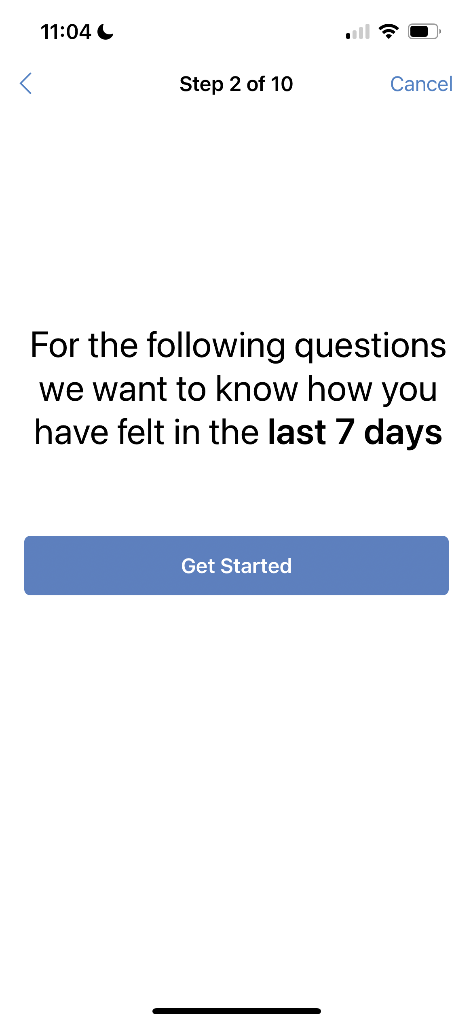
**

**
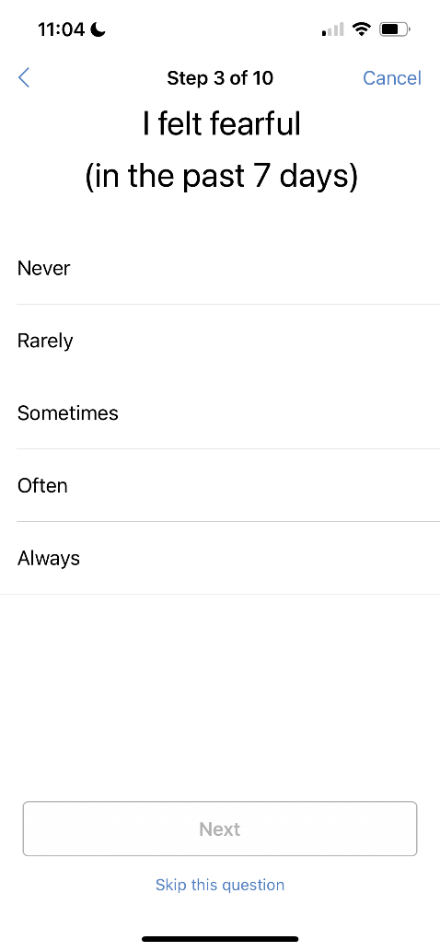

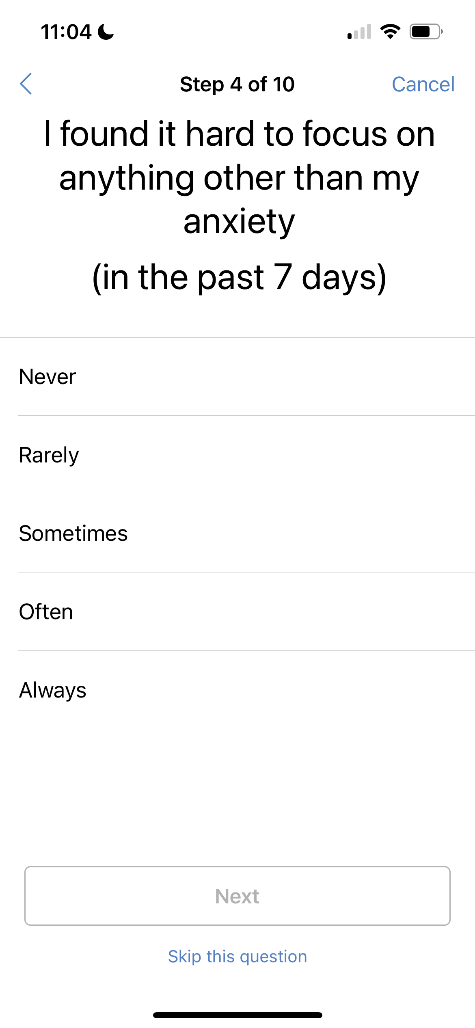

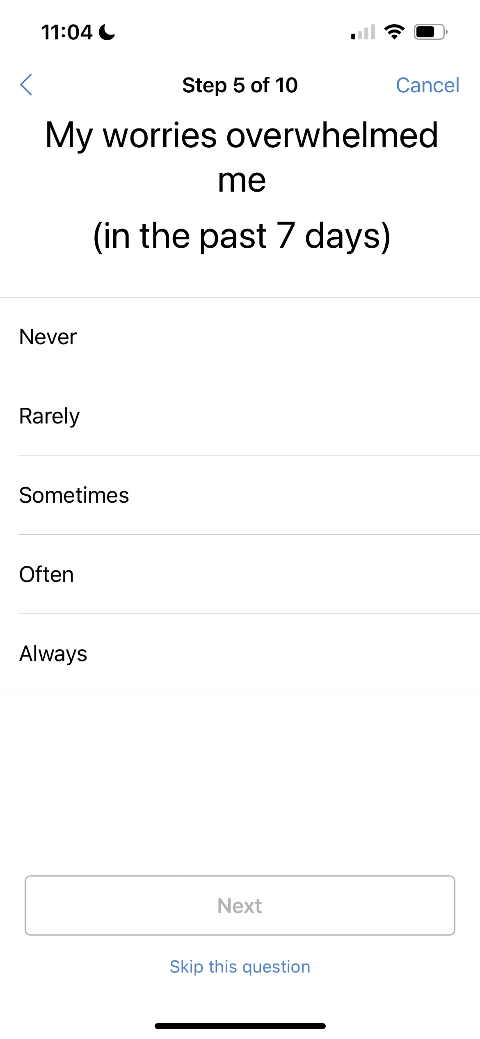

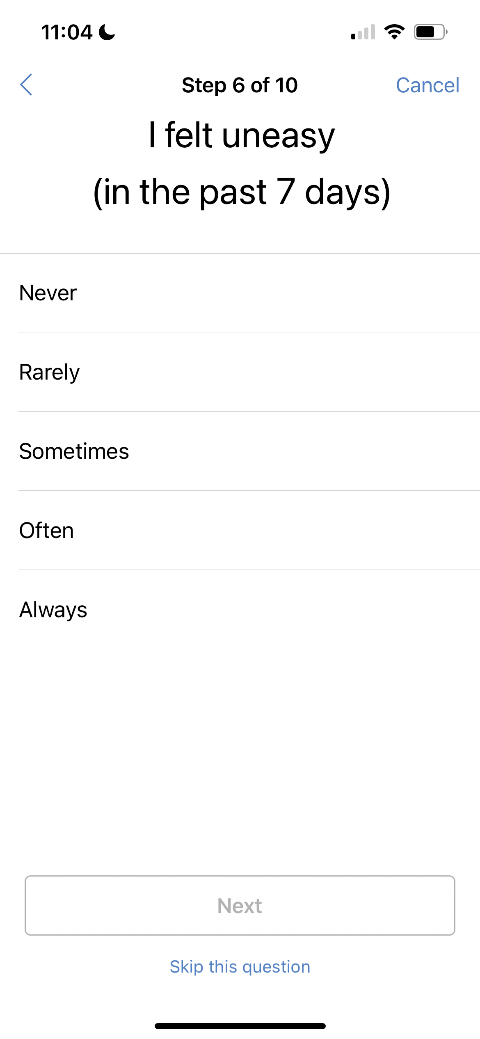

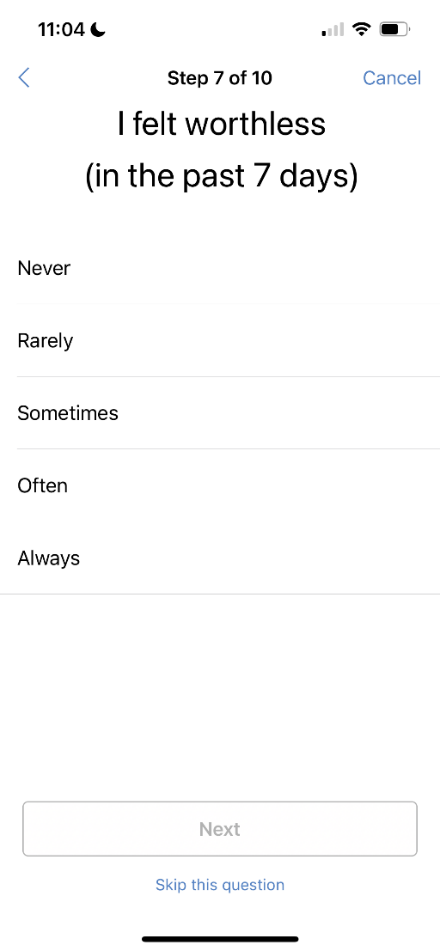

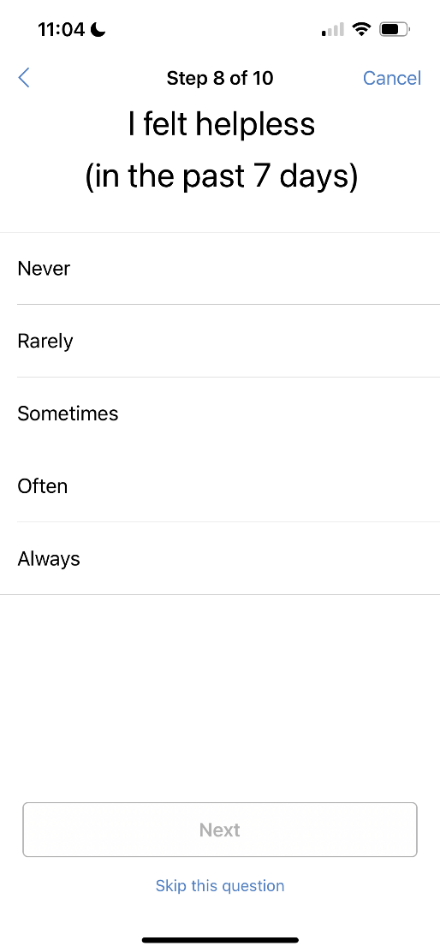
**

**
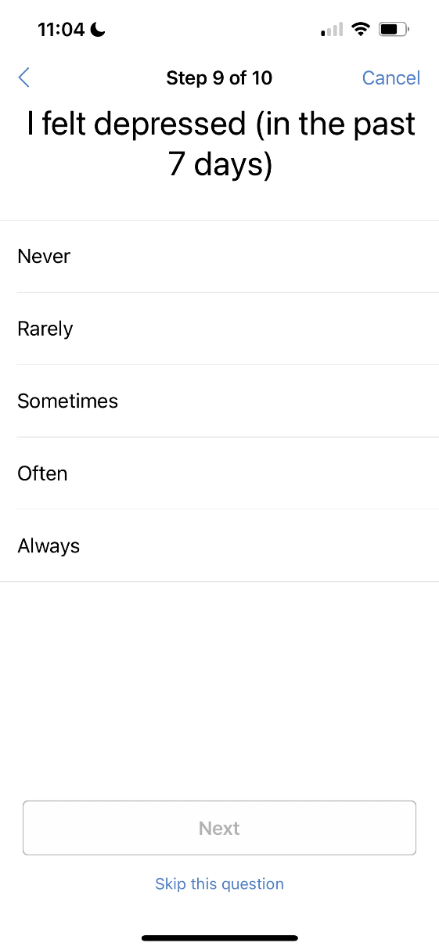

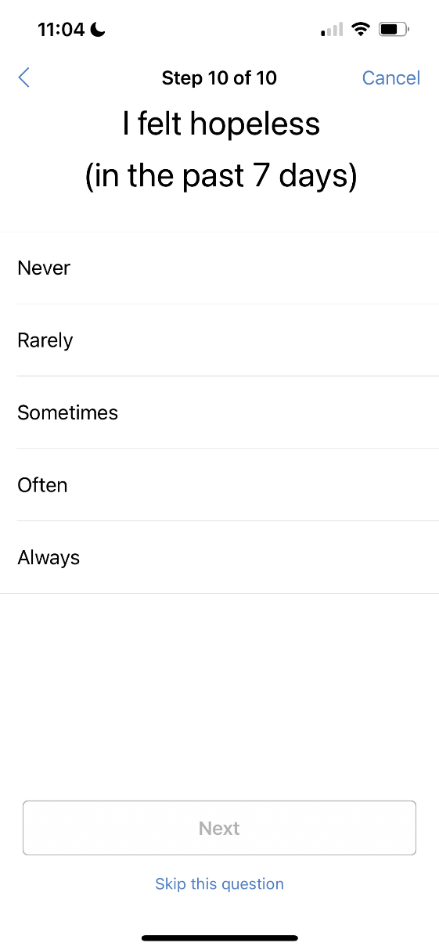

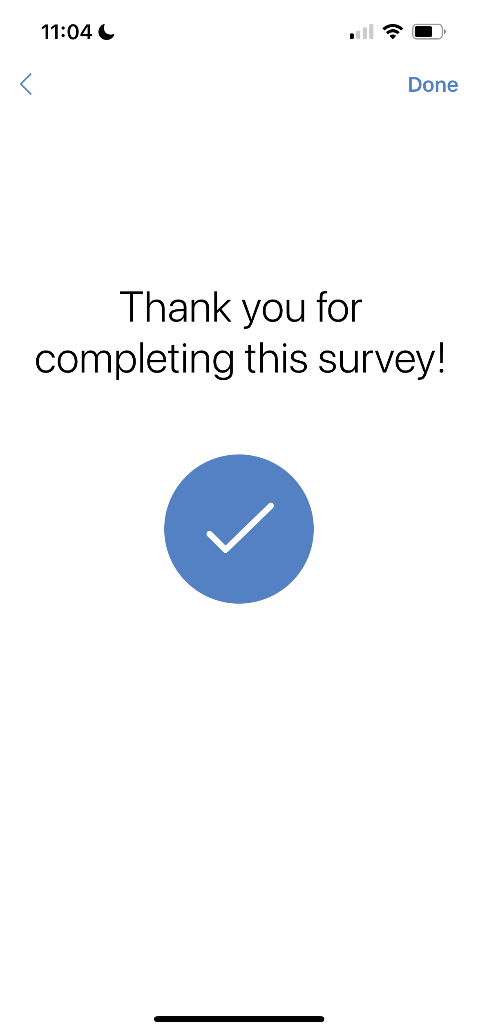
**

**Mood 2 Survey:**


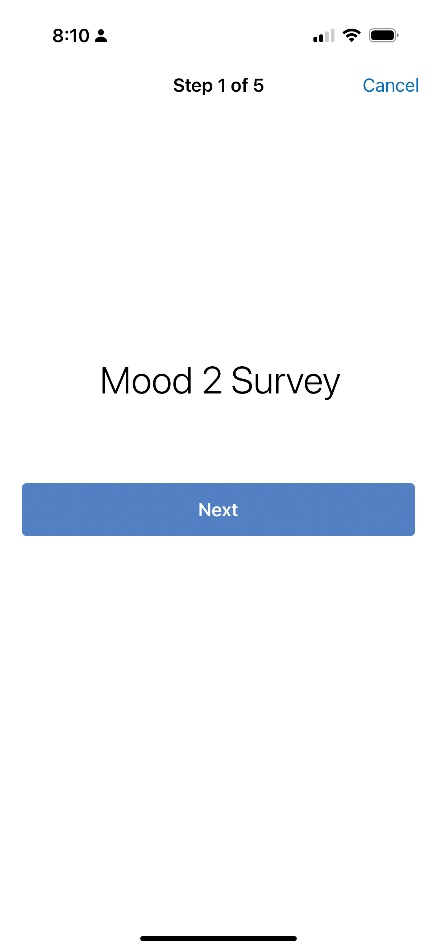

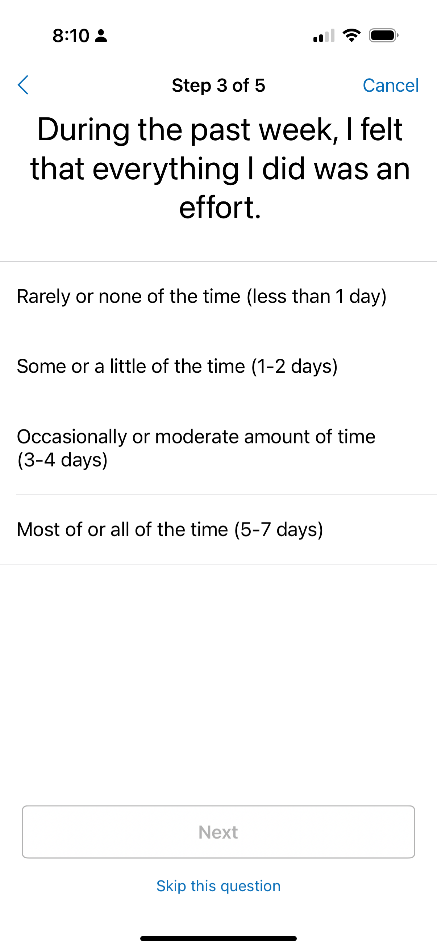

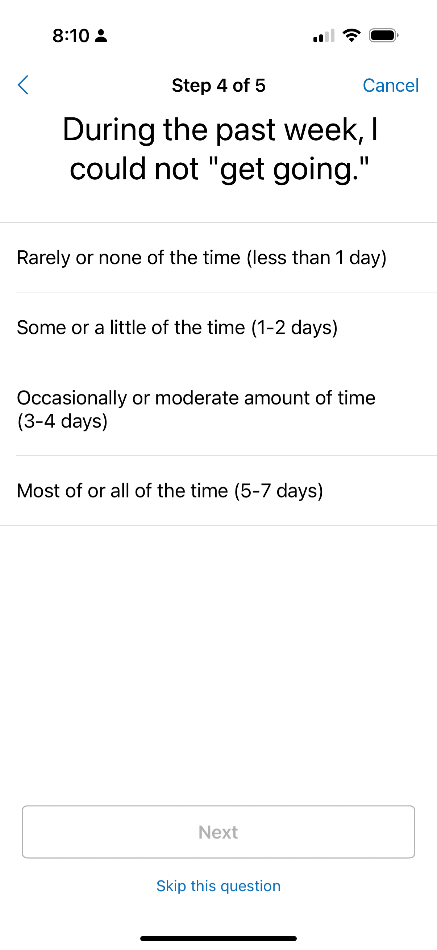


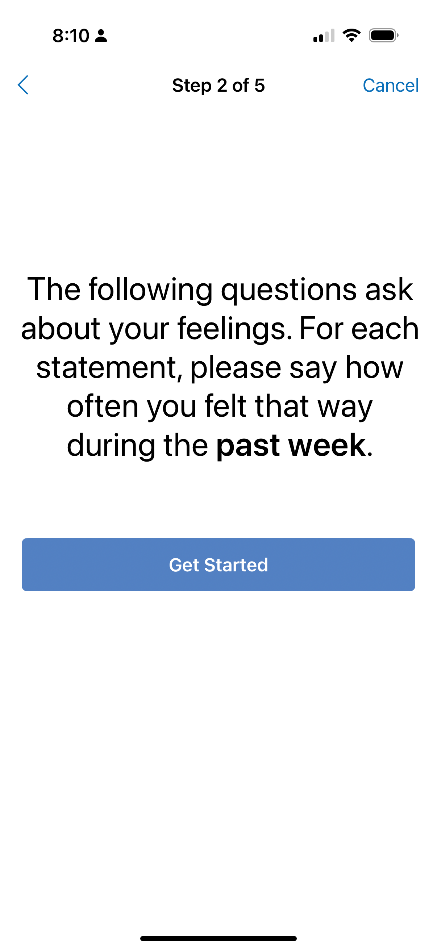

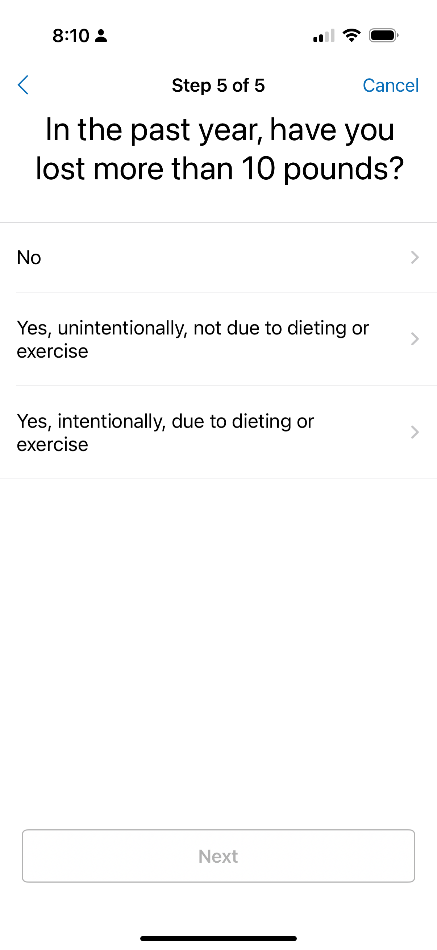

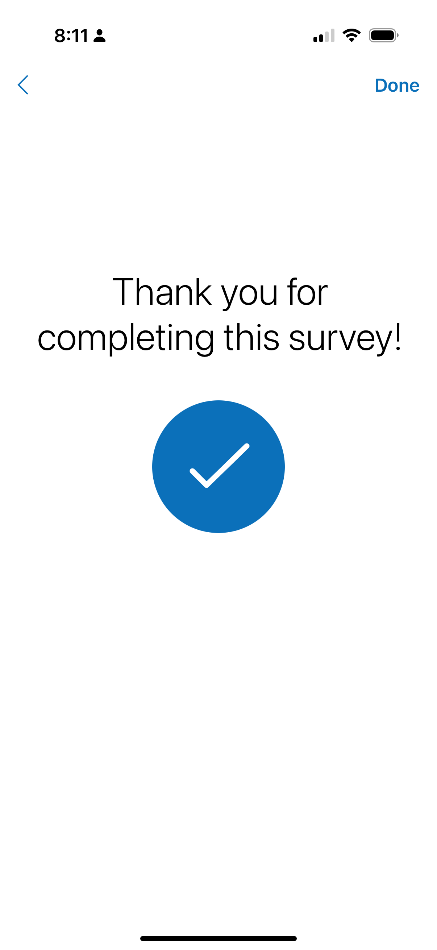


**Pain Q:**

**
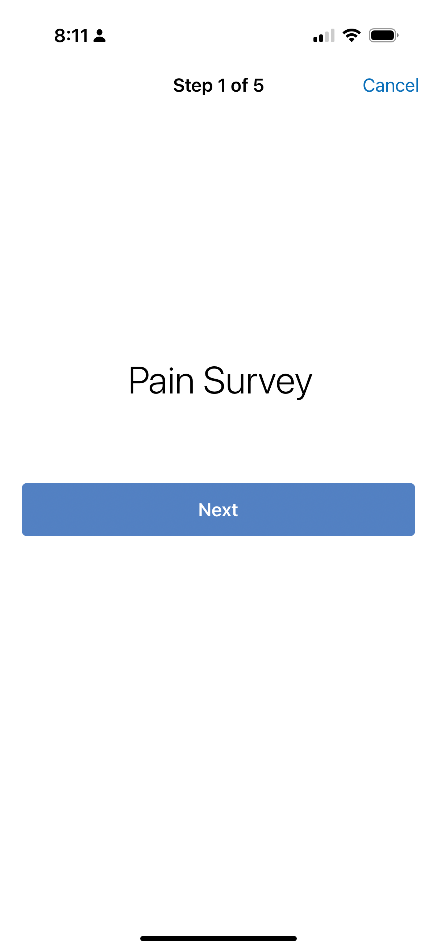

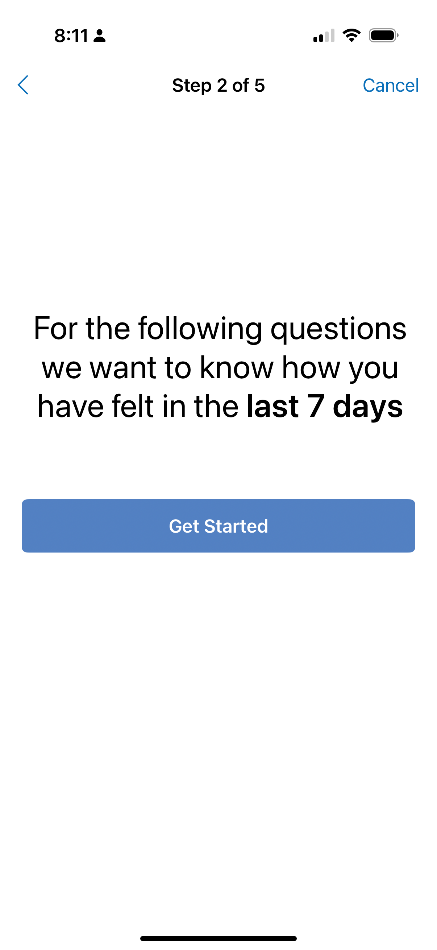

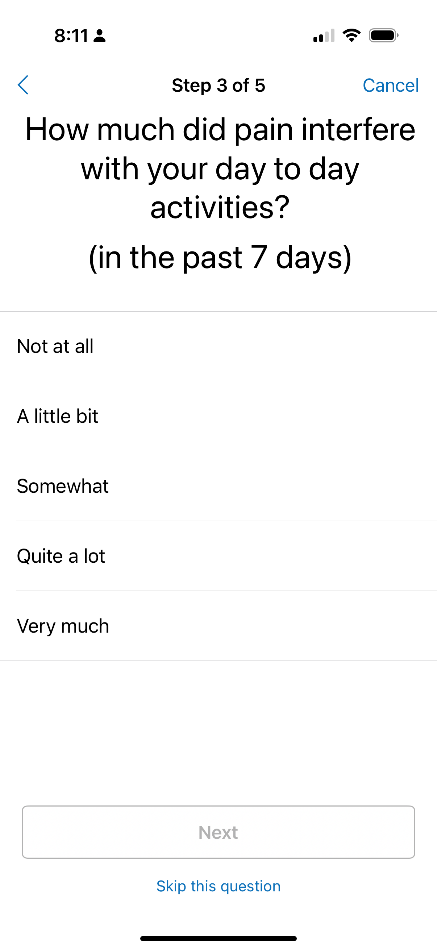
**

**
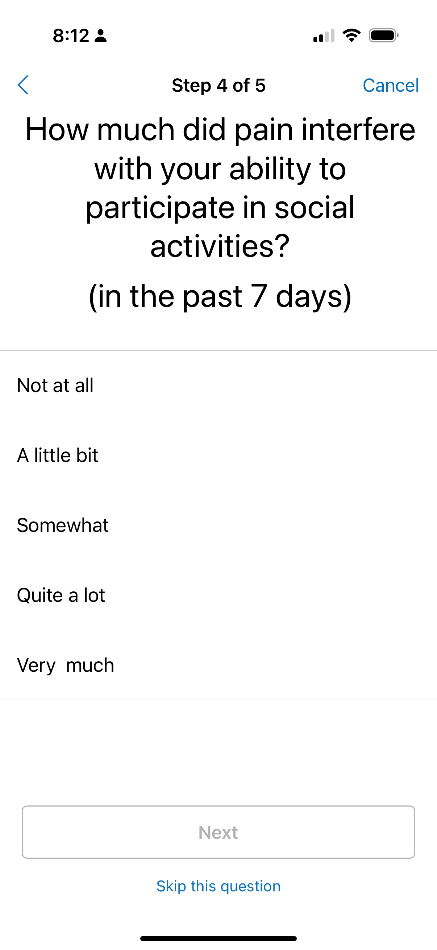

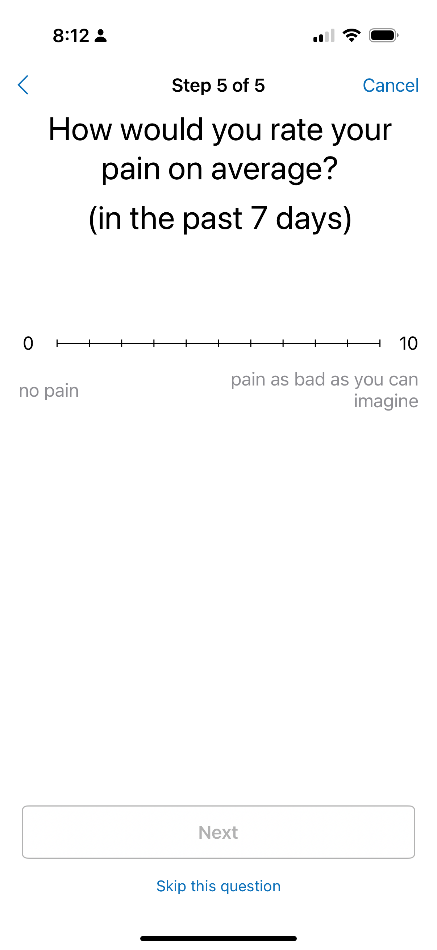

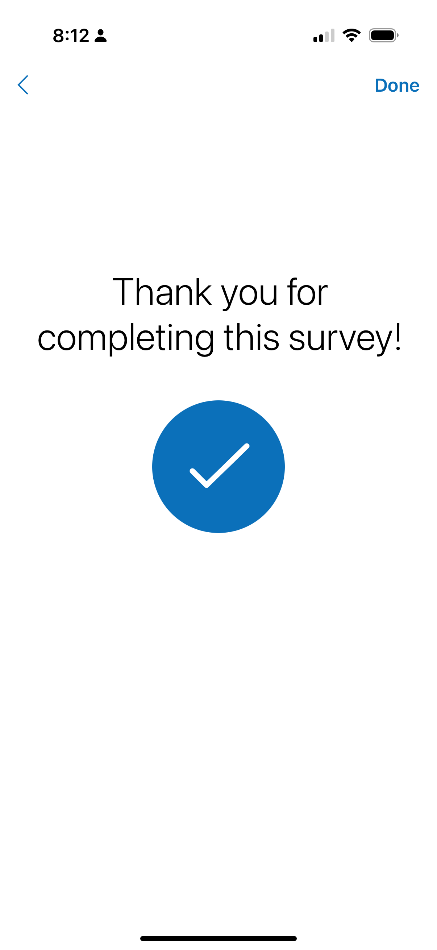
**

**Body Pain Map:**

**
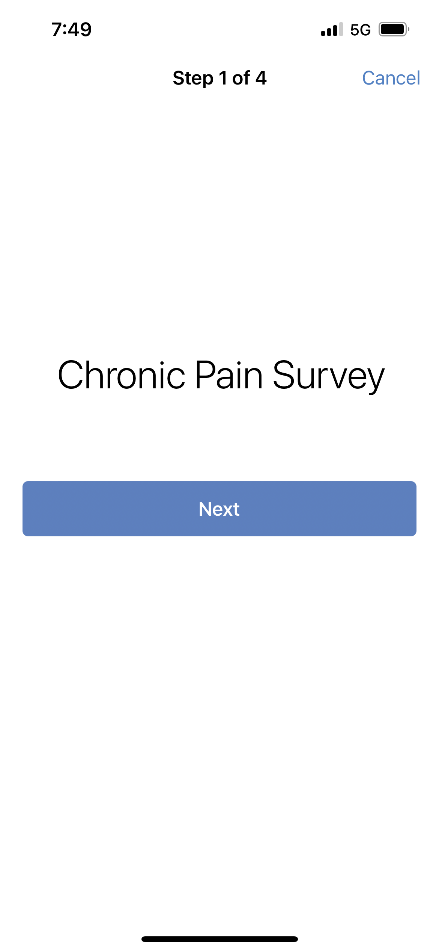
**

**
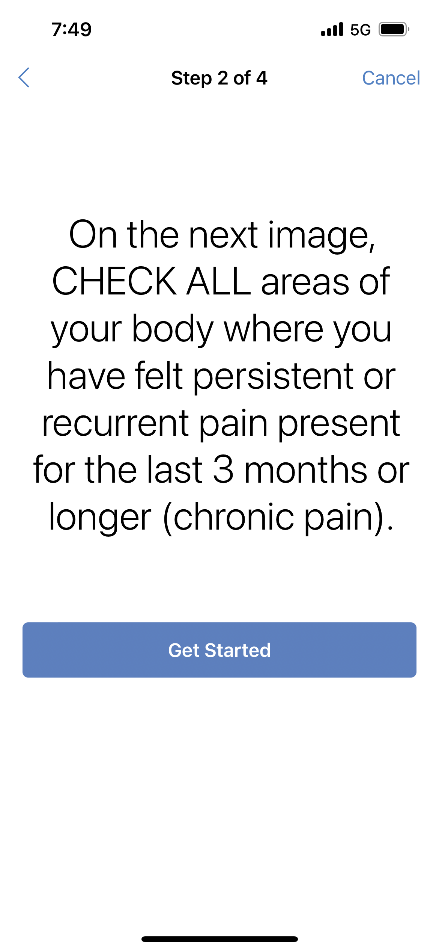

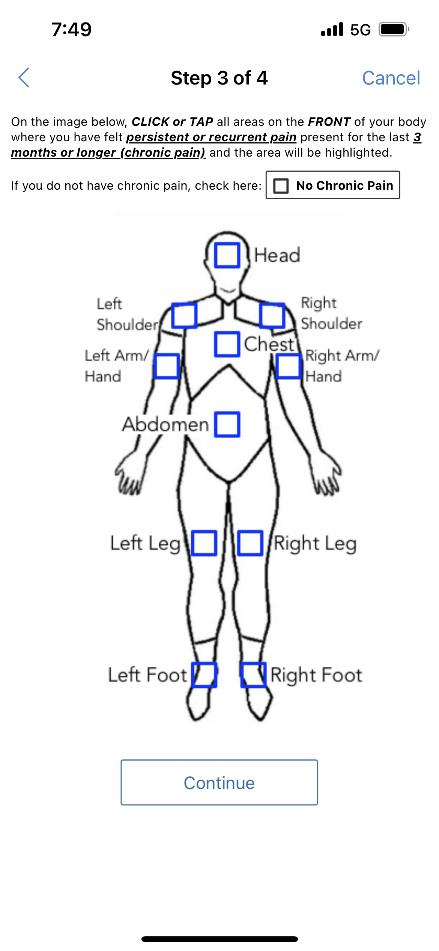

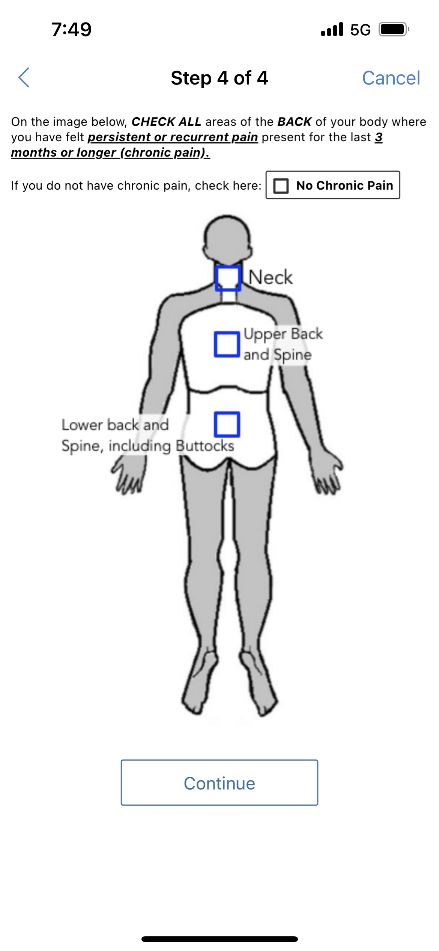

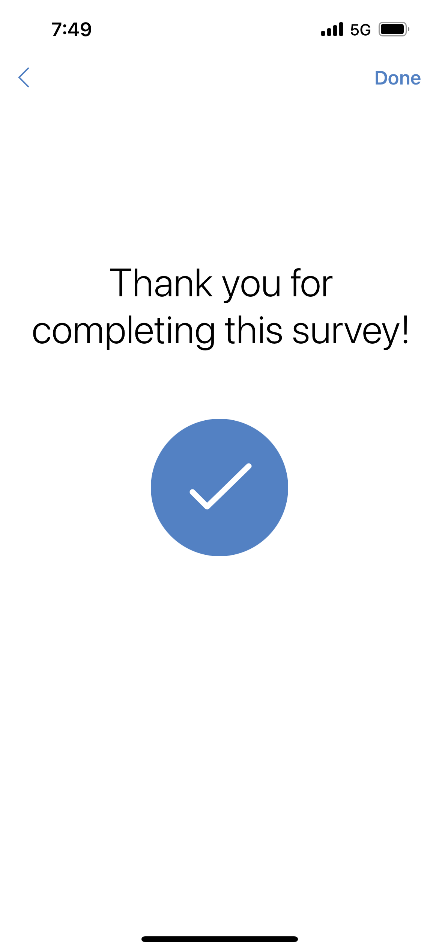
**

**Physical Function Survey:**

**
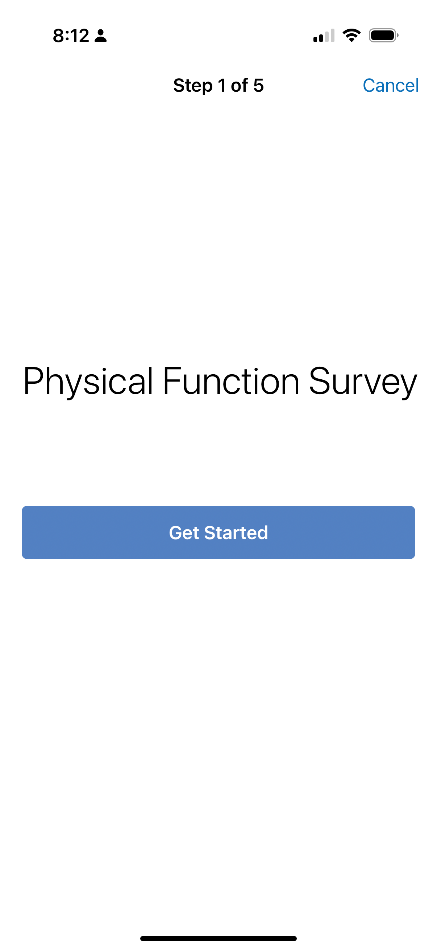

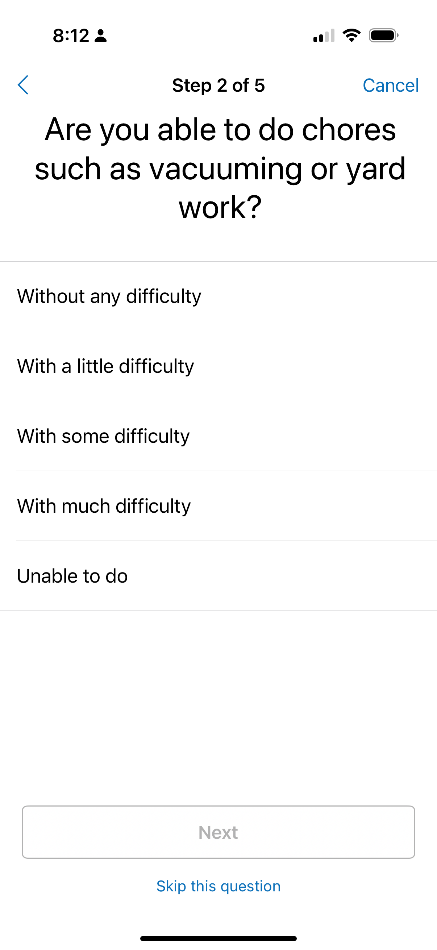

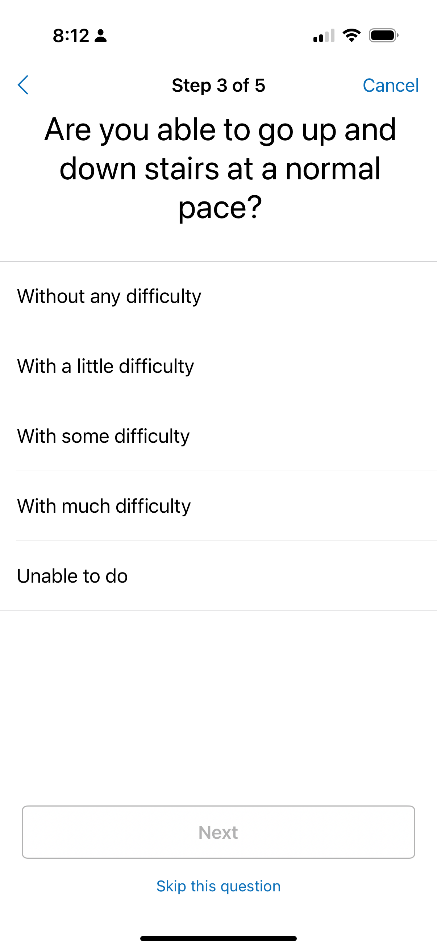
**

**
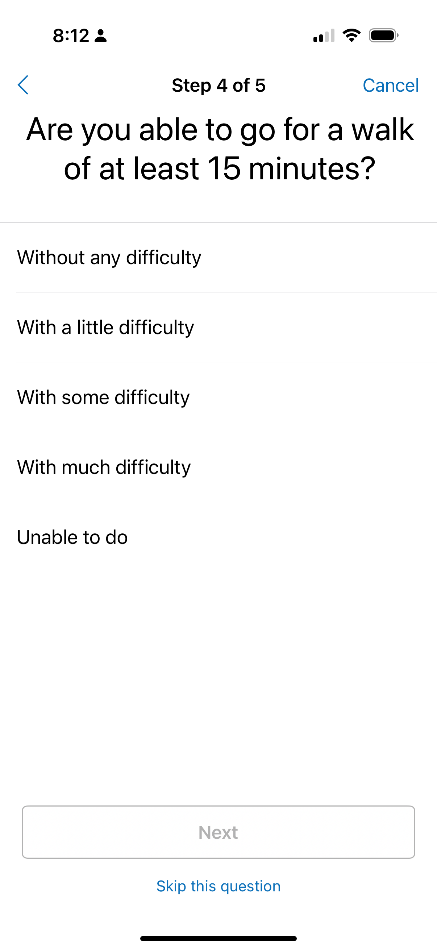

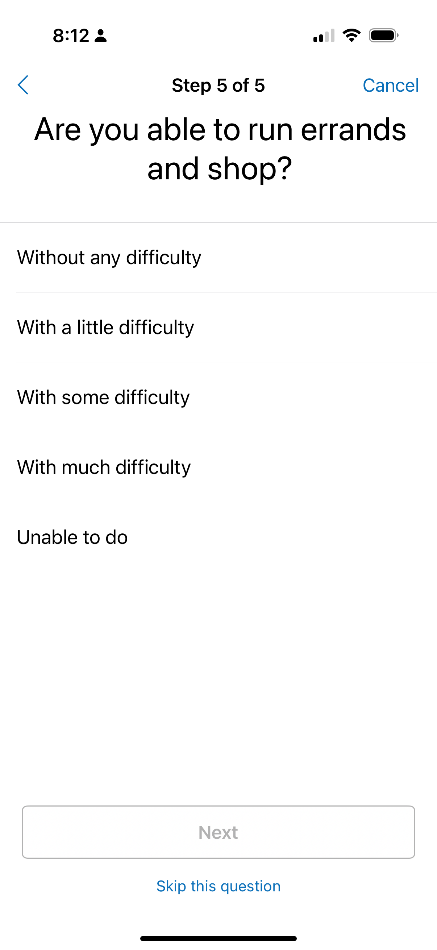

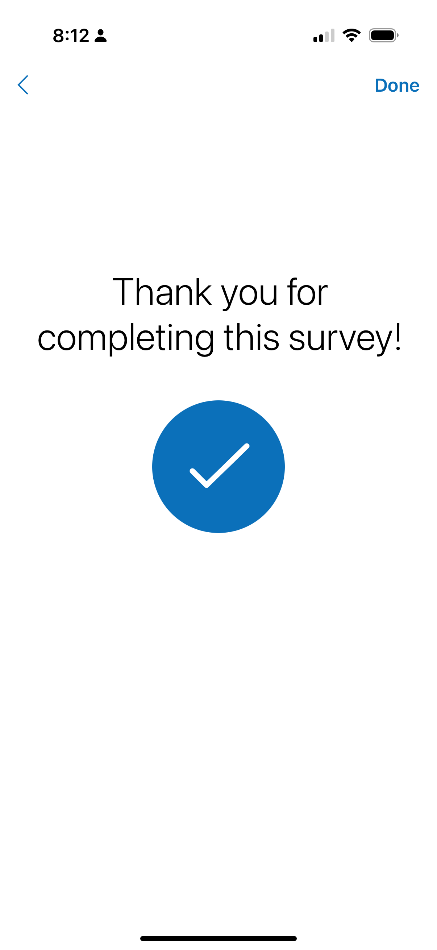
**

**Sleep Survey:**


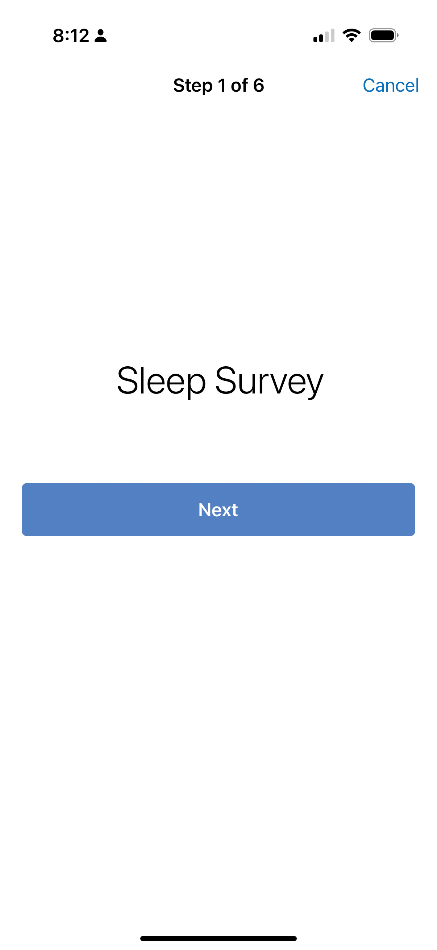

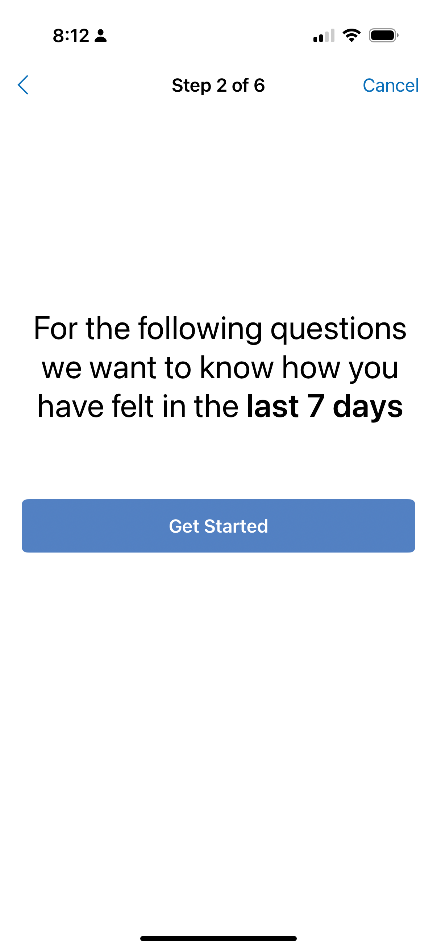

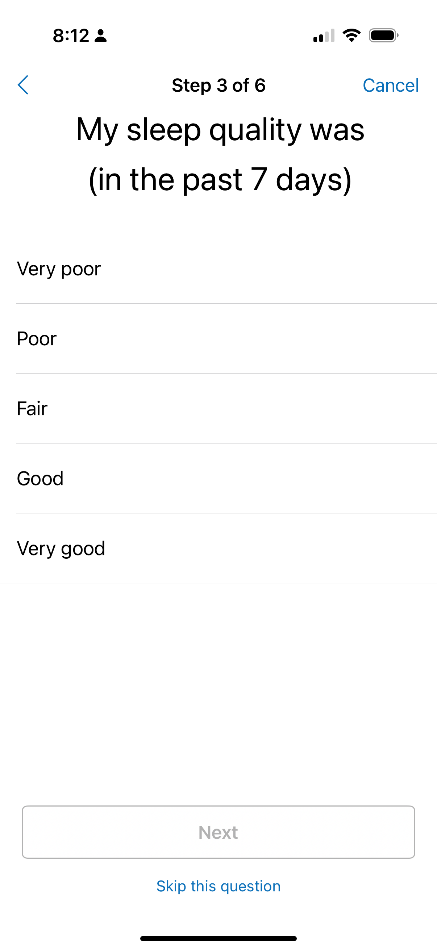


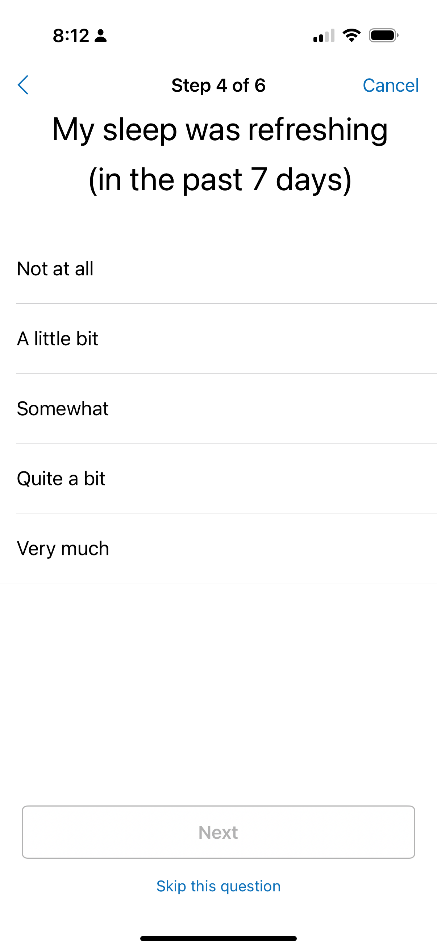

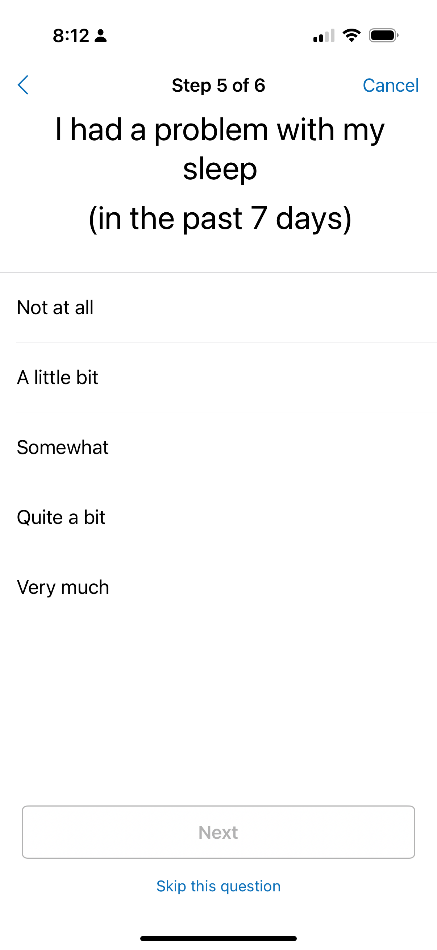

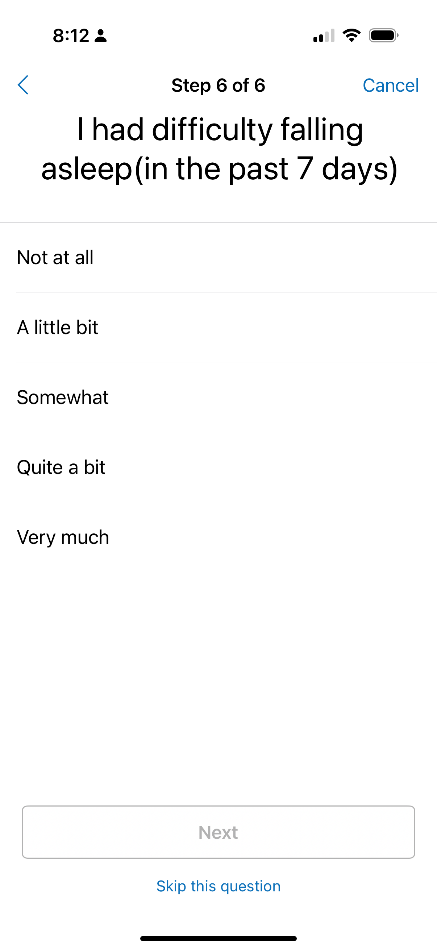

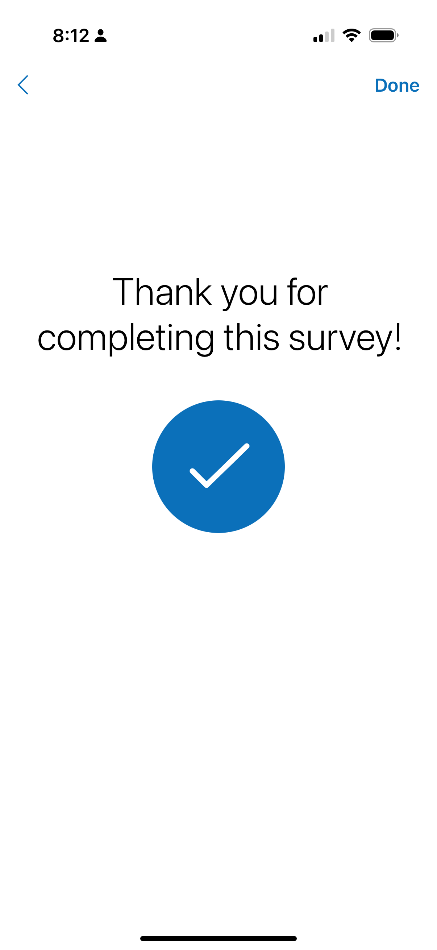


**Fatigue Survey:**


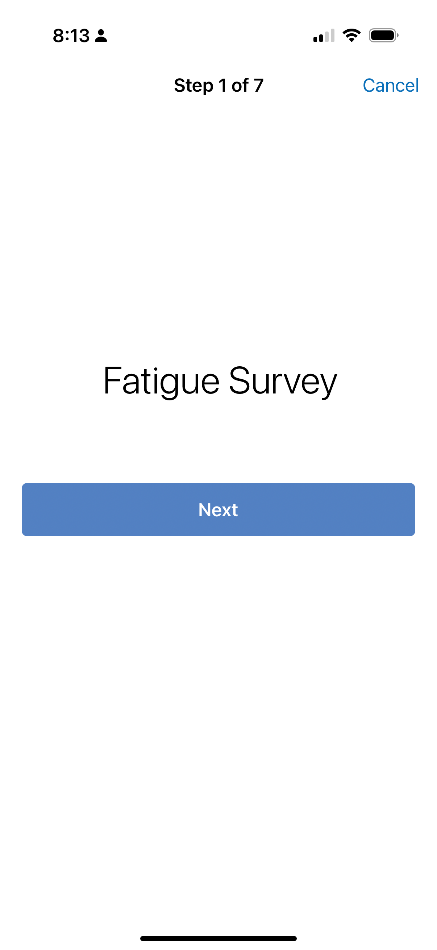

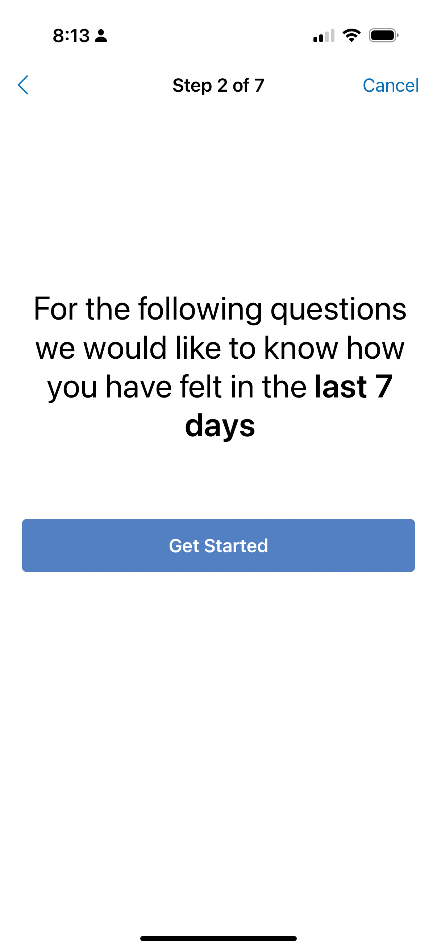

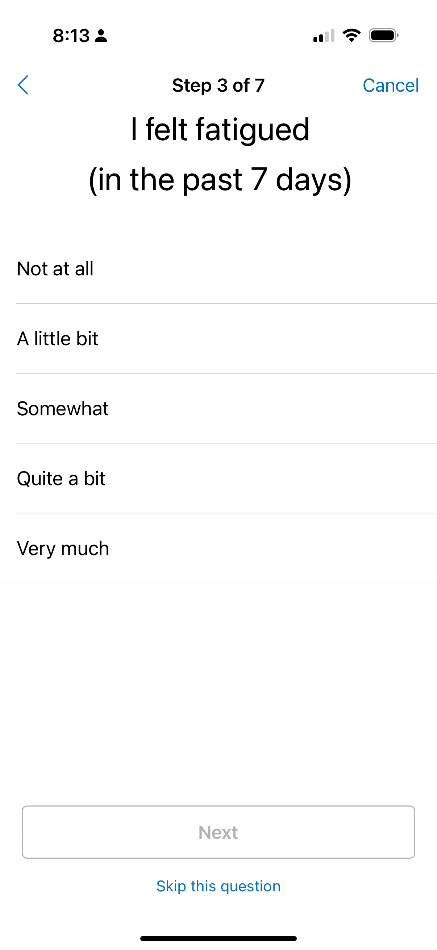


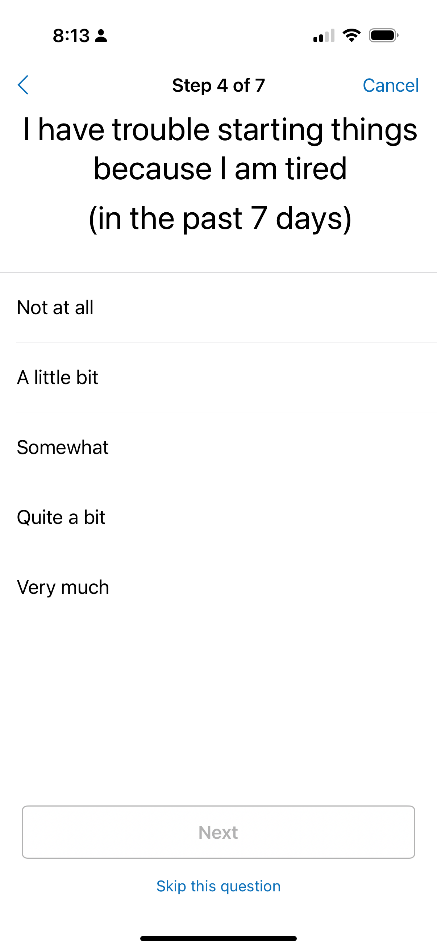

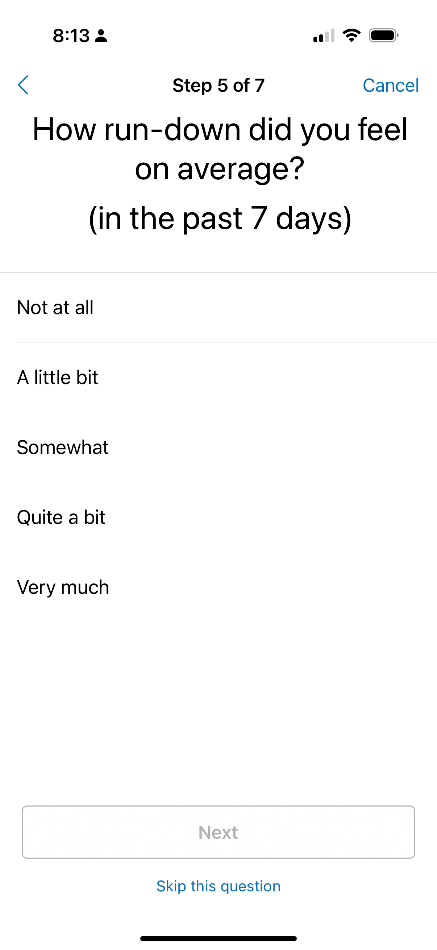

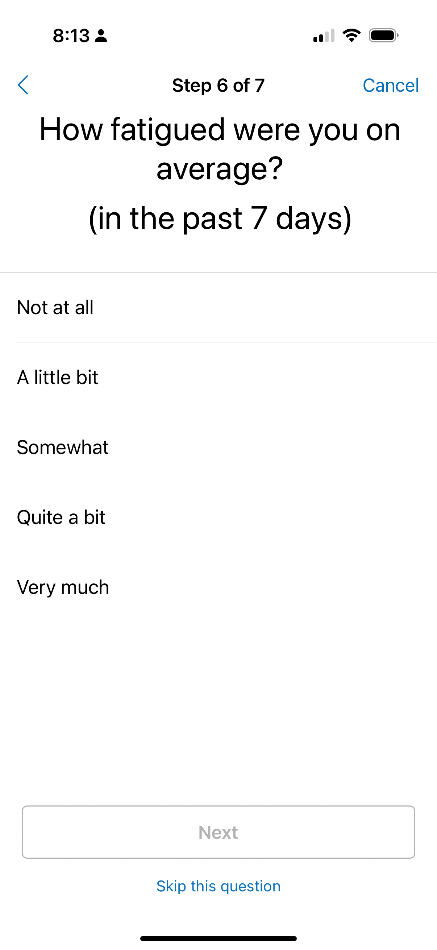

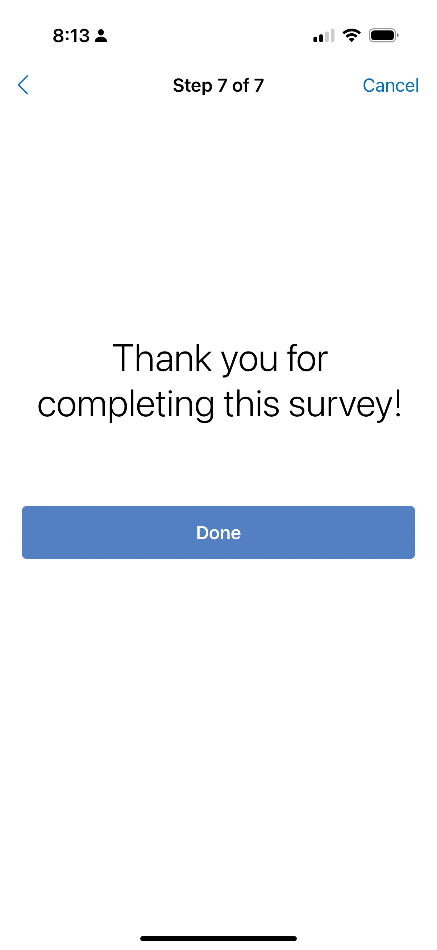


**Mobility outside The Home Survey:**


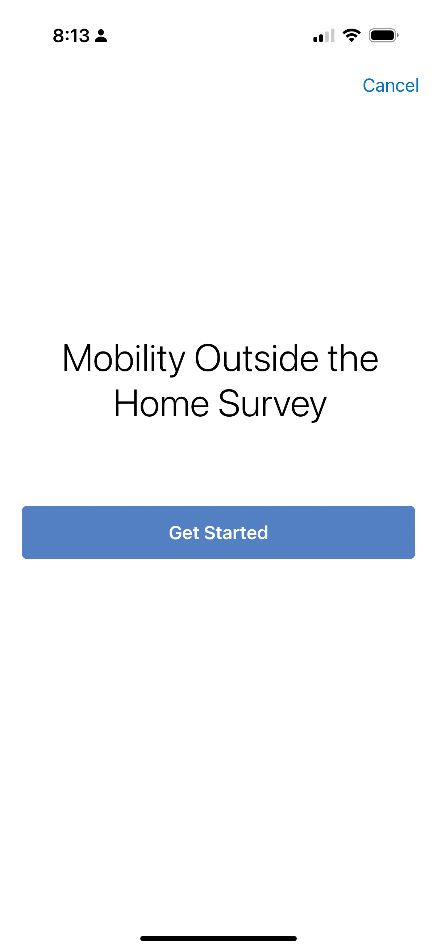


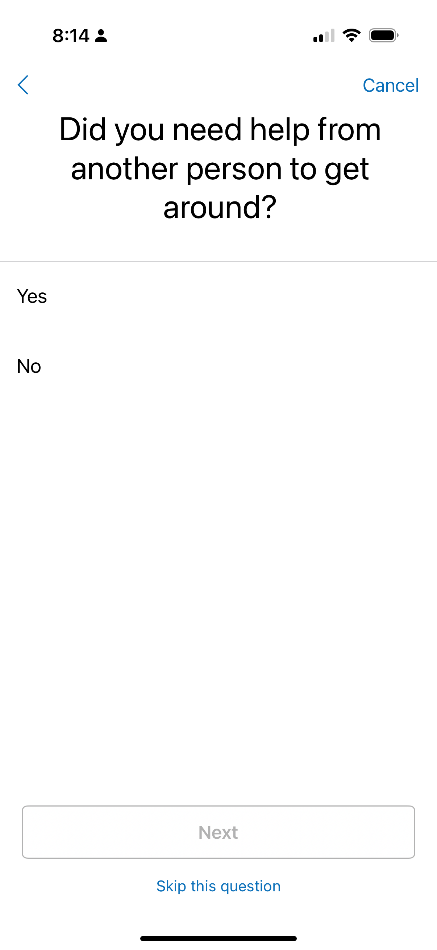

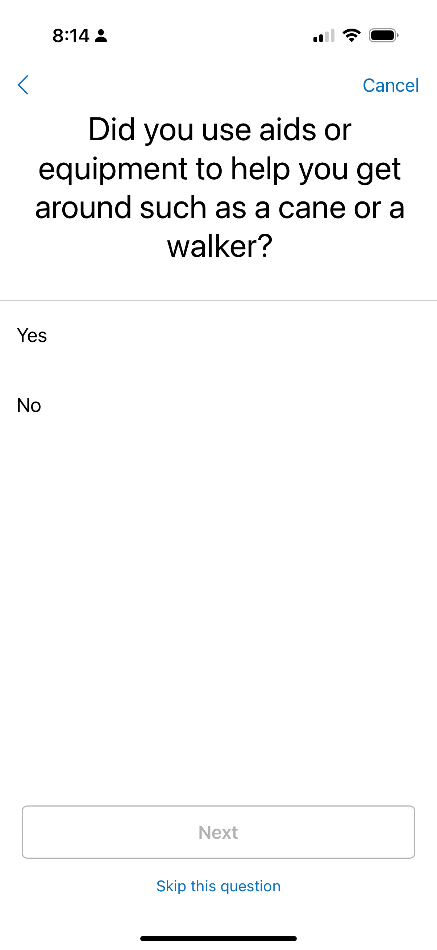

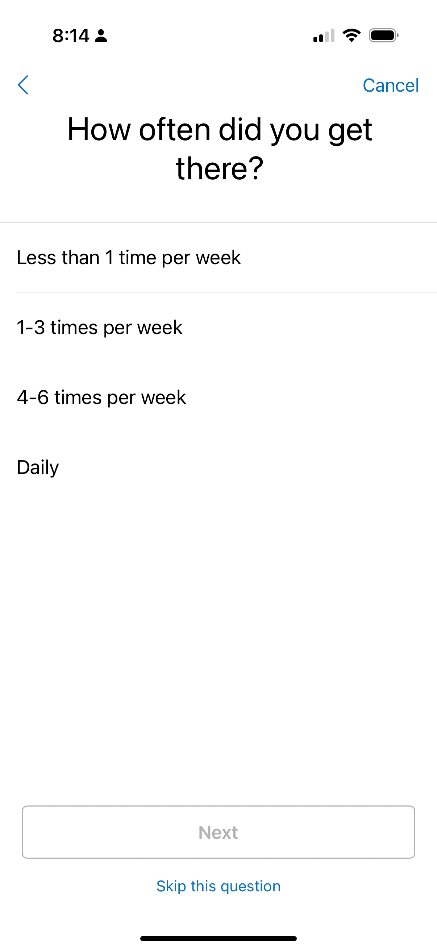

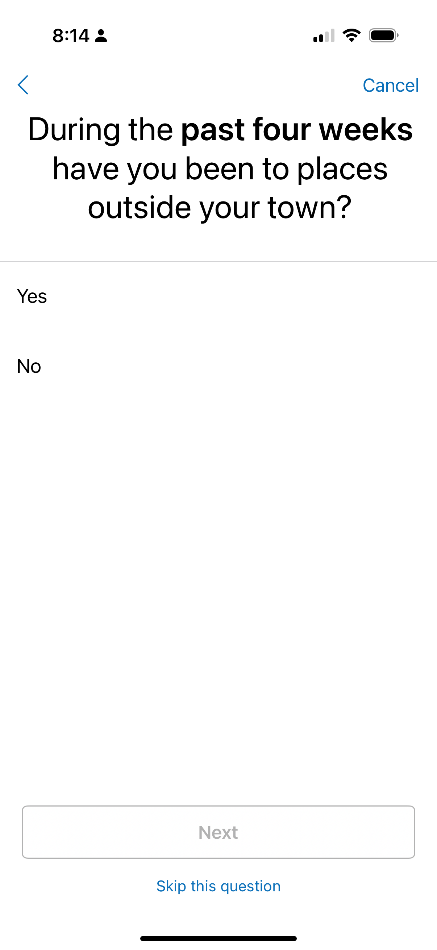

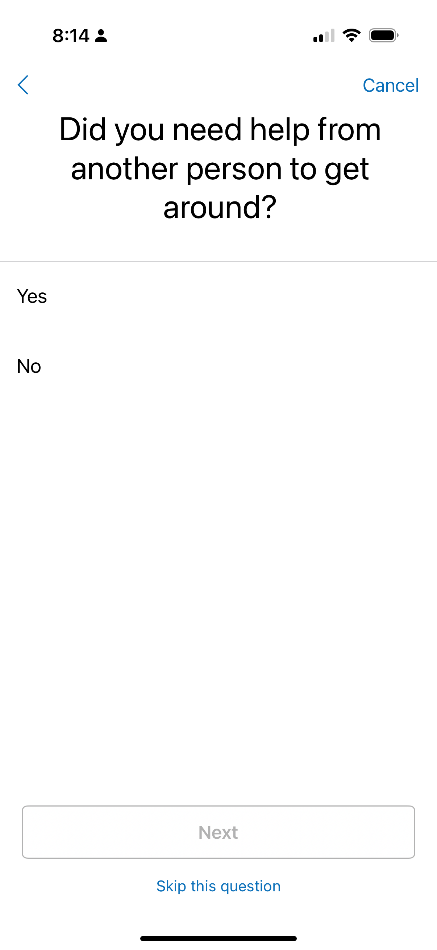

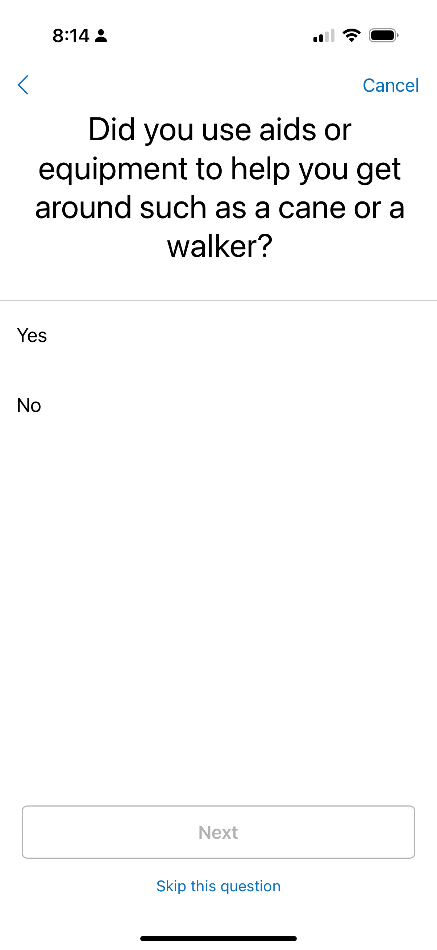

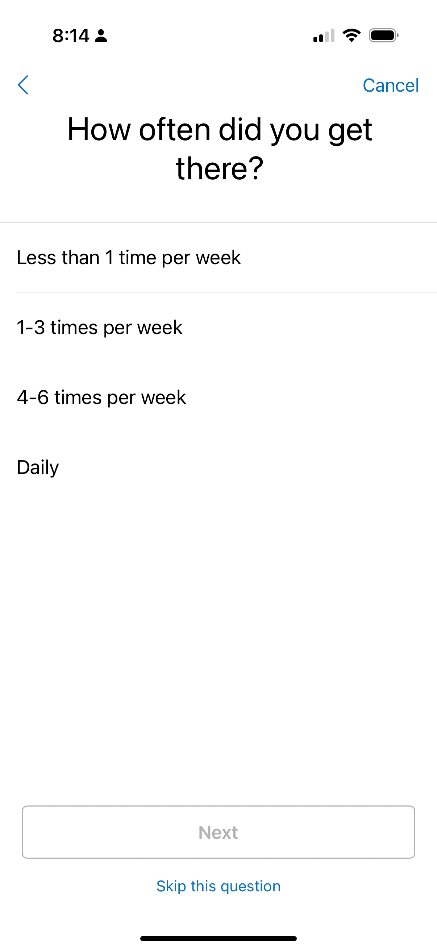

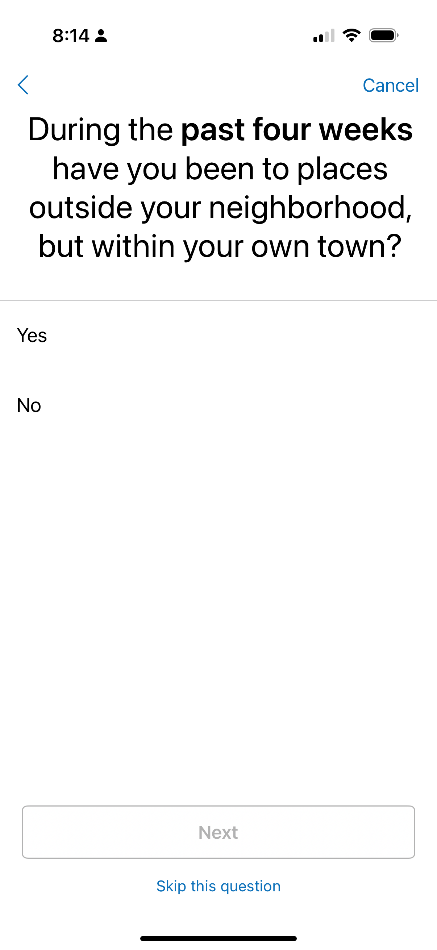

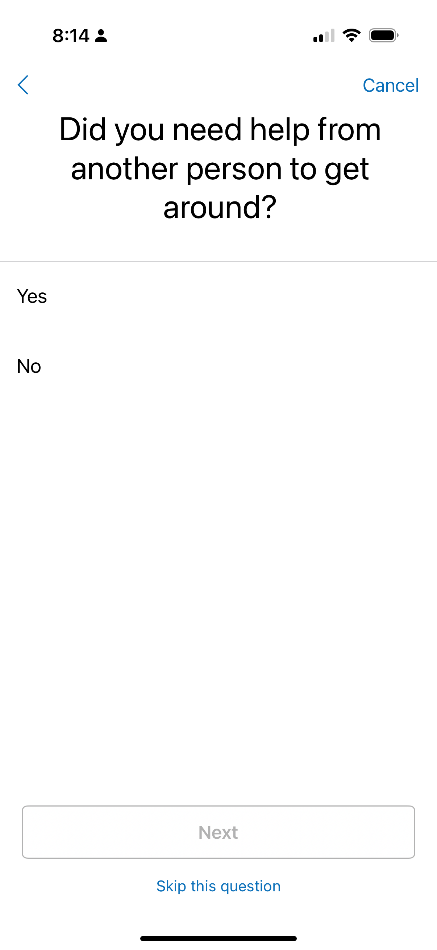

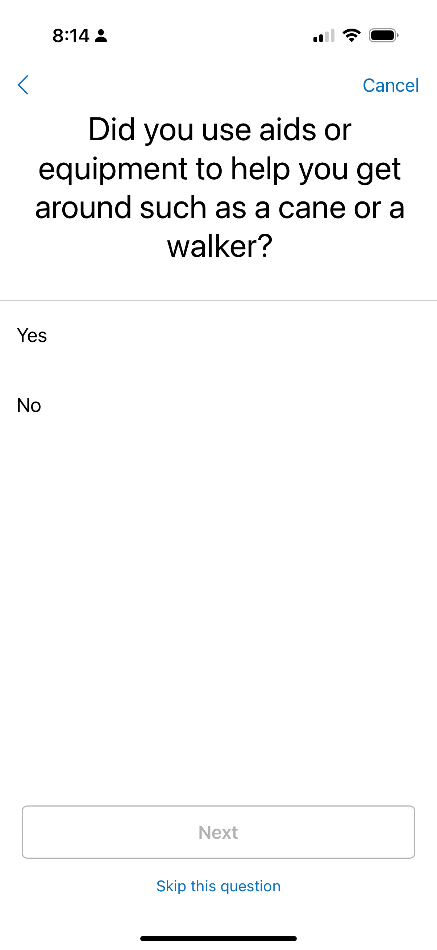

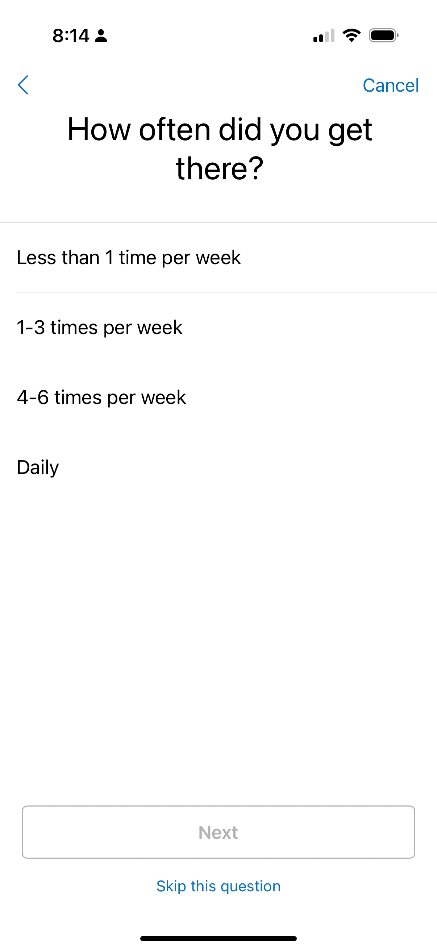

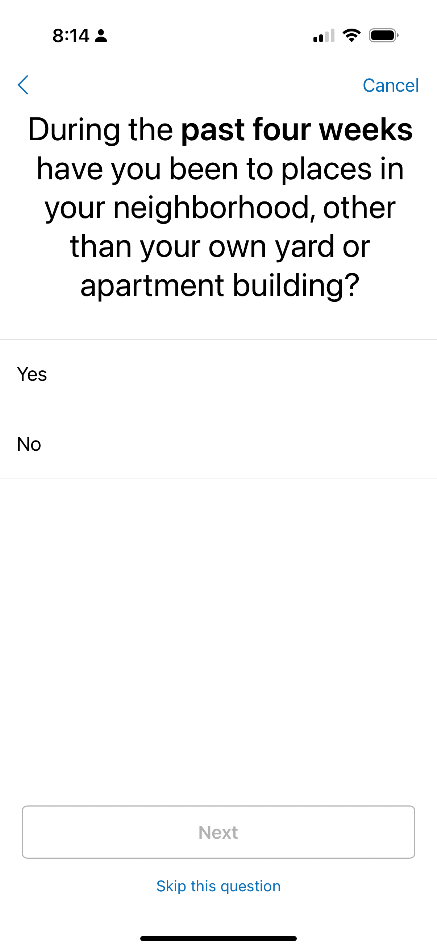

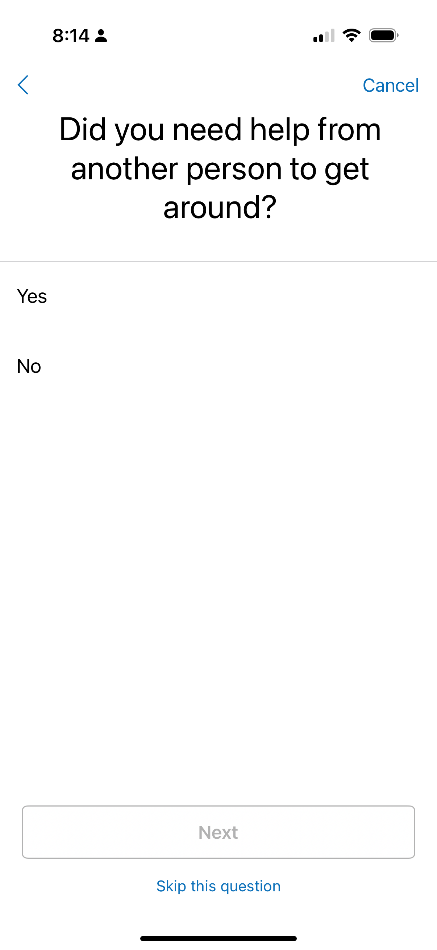

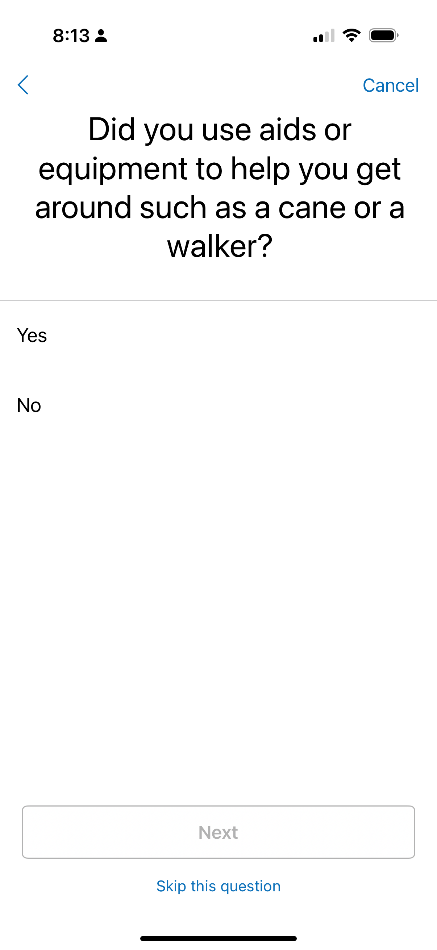

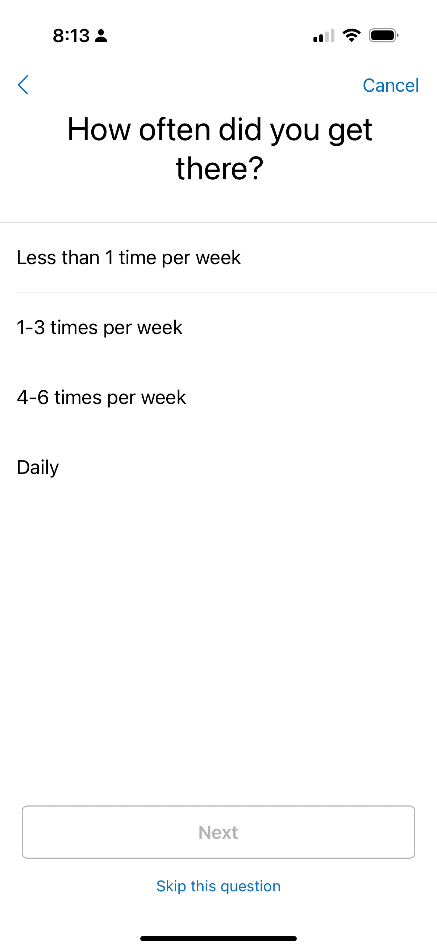

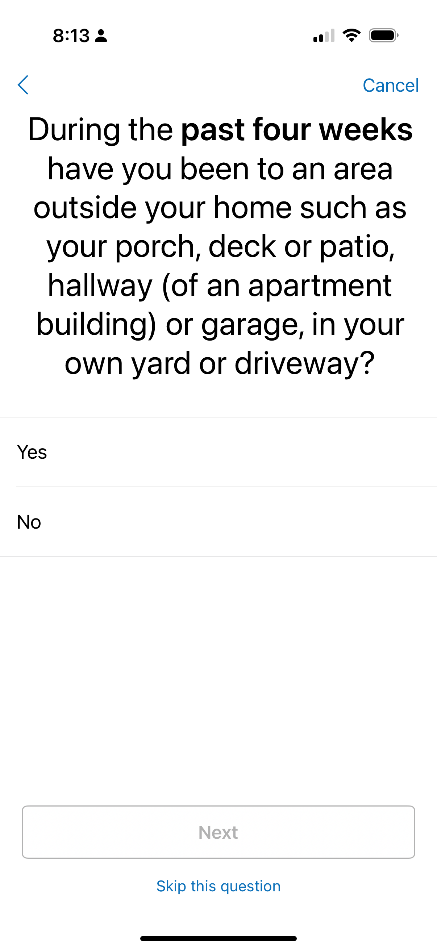

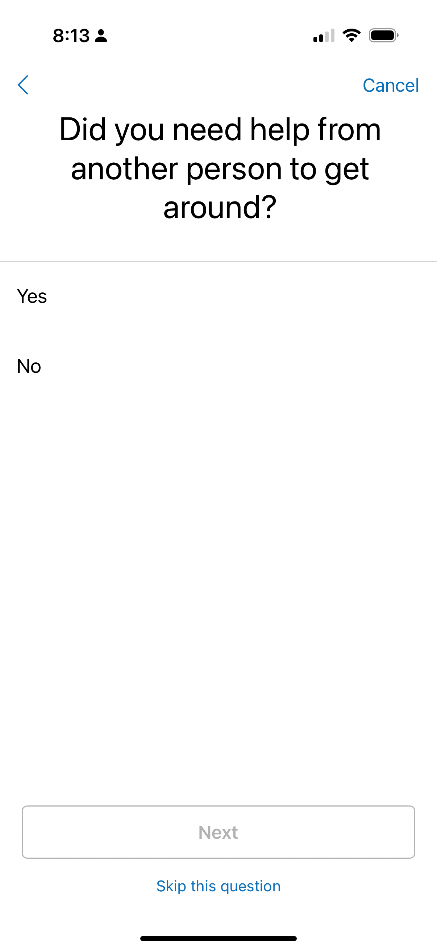

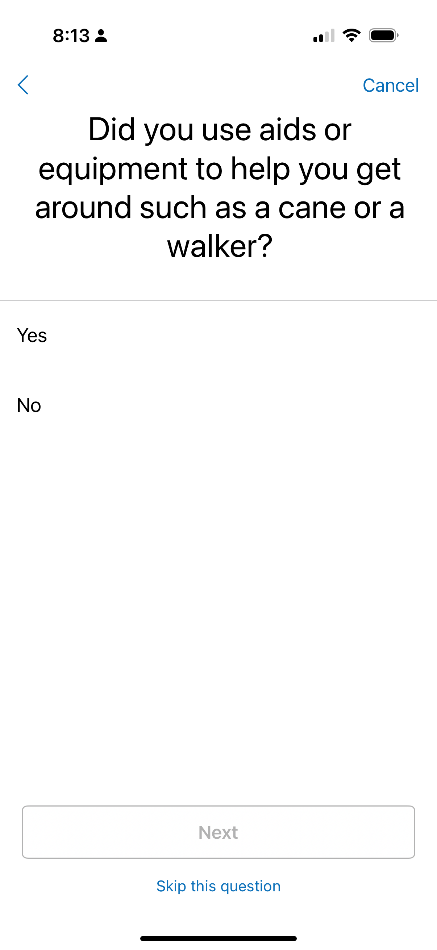

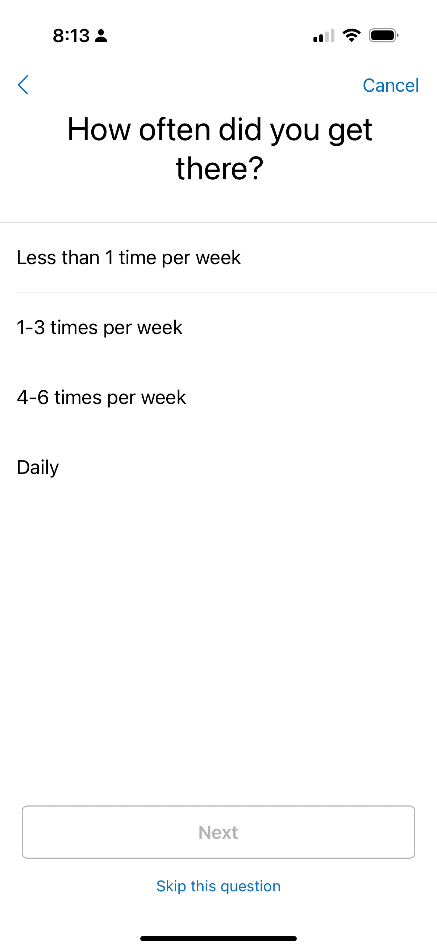

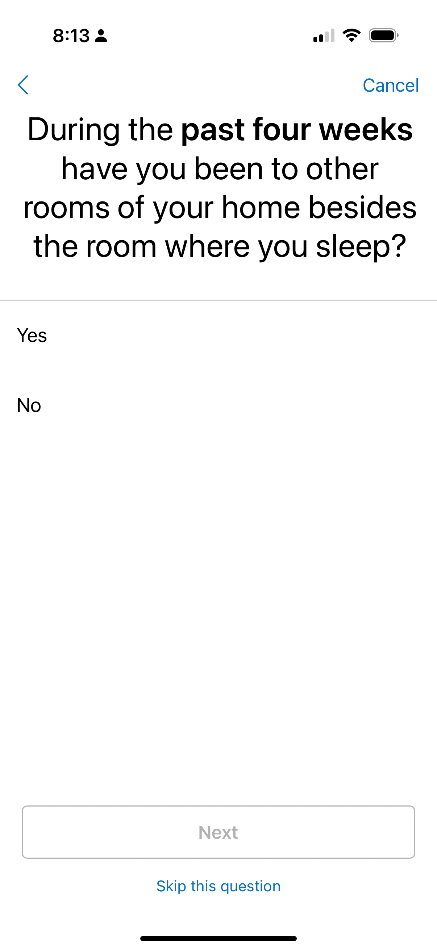

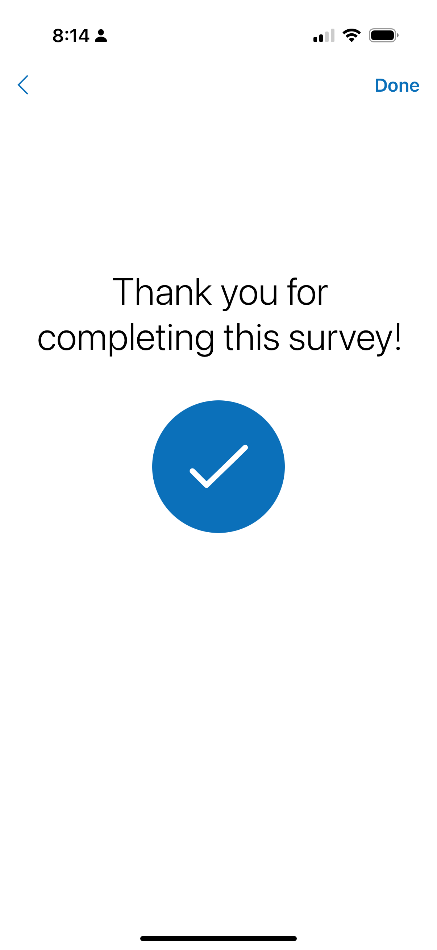


**Rapid Assessment of Physical Activity:**

**
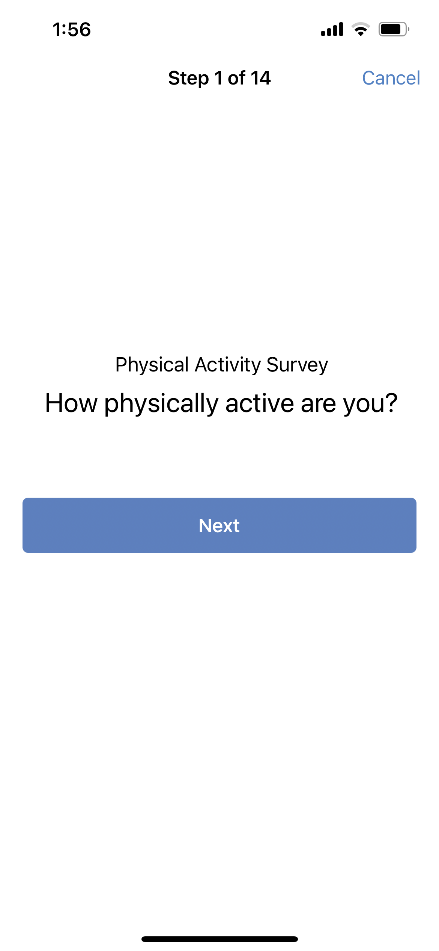

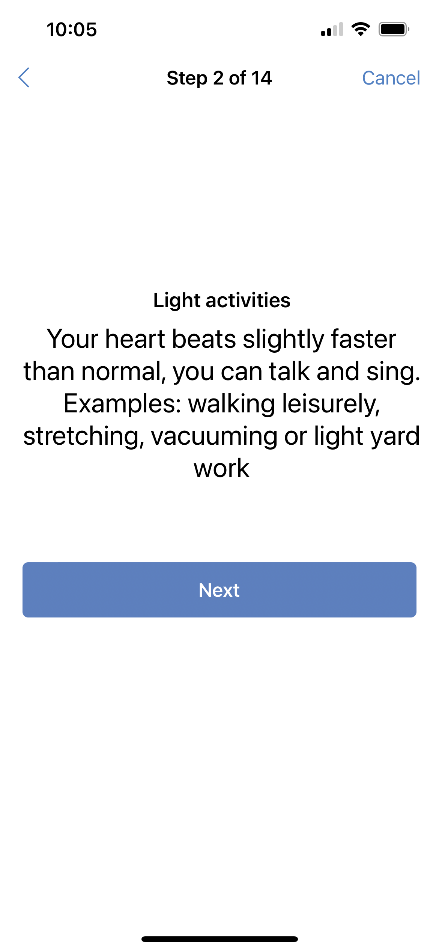

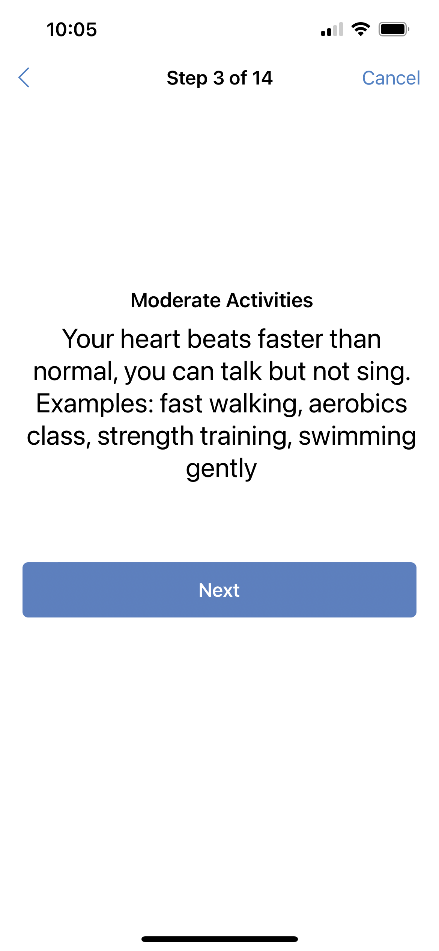
**

**
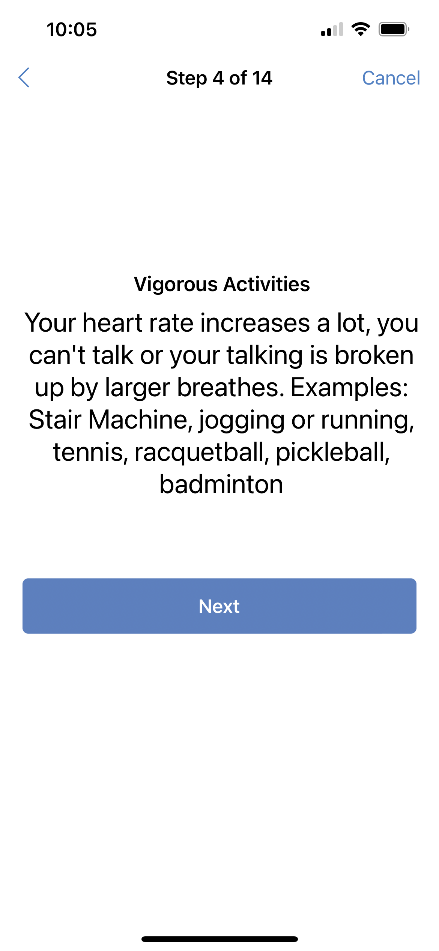

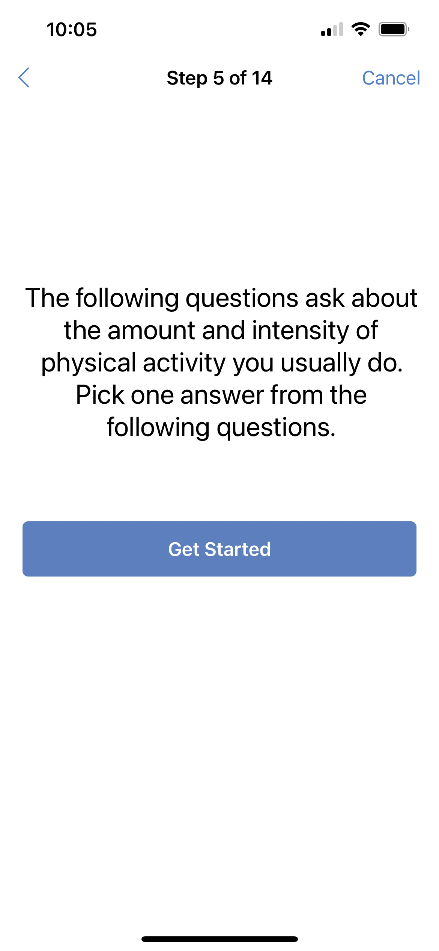

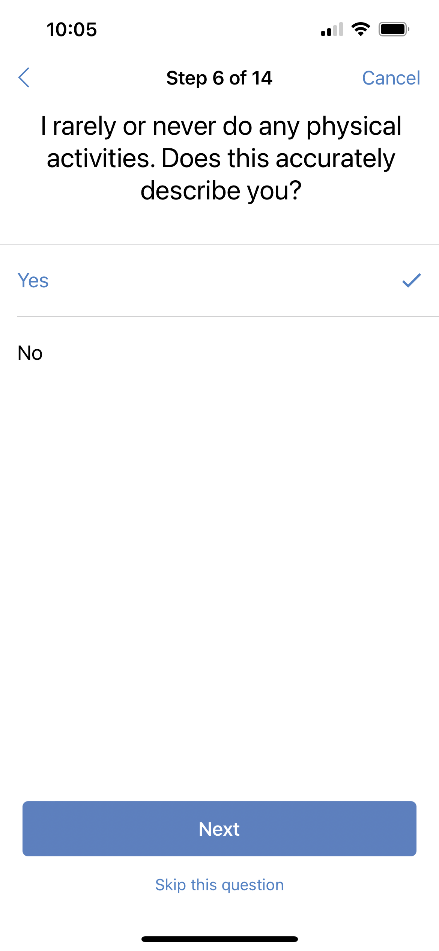

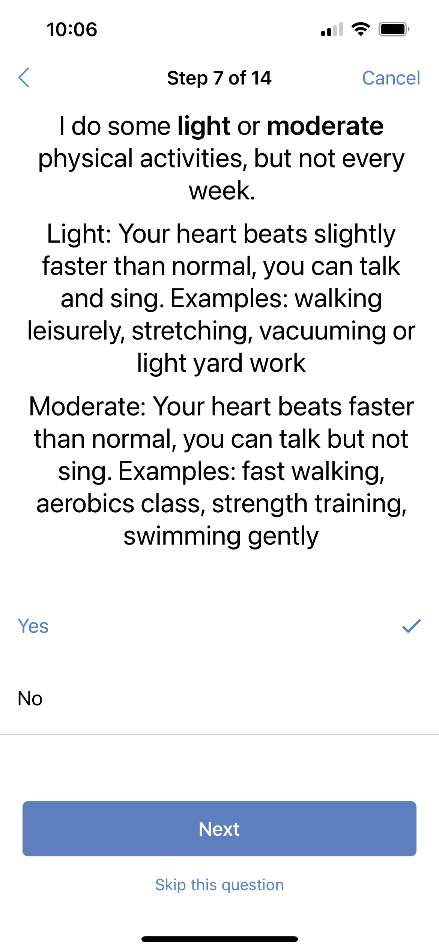

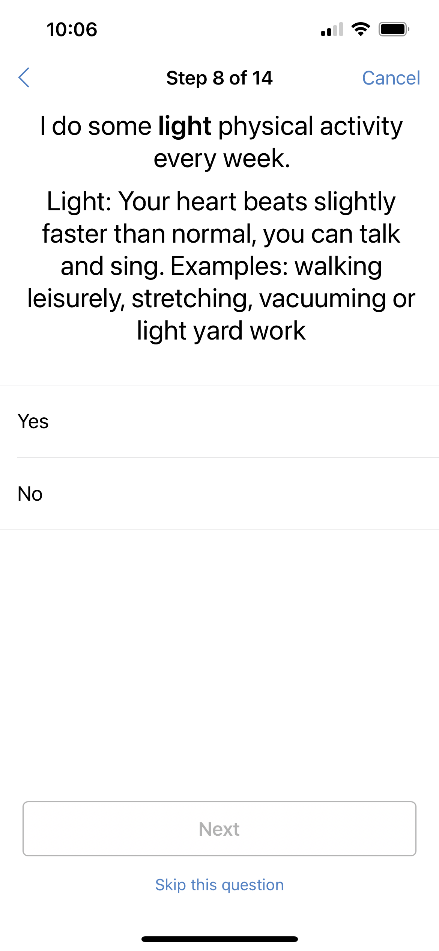

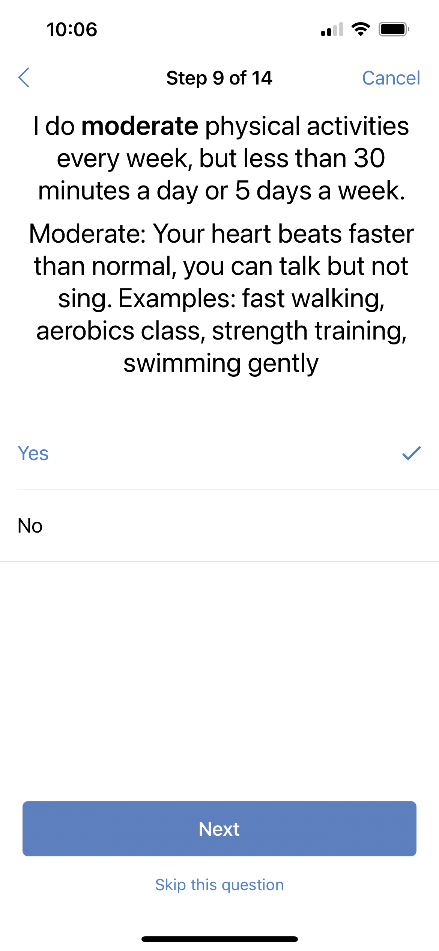

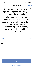

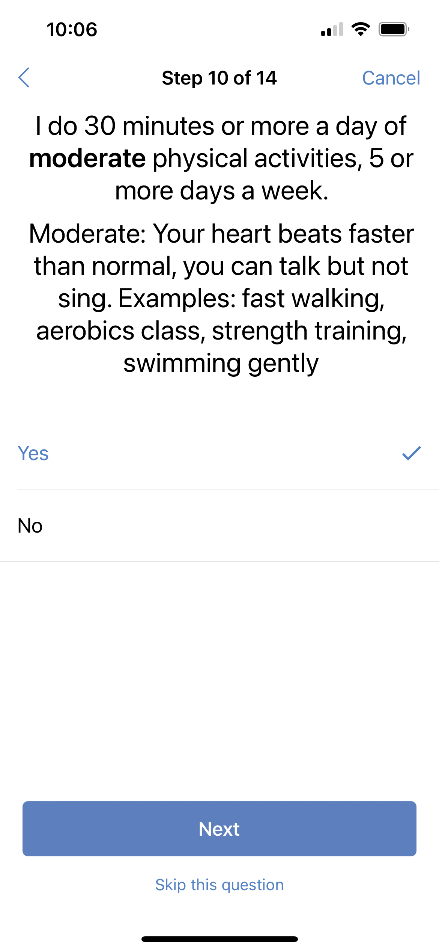

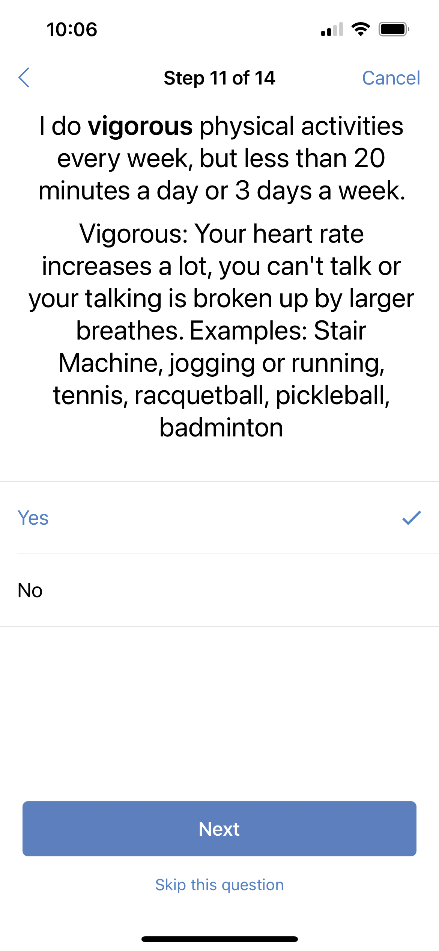

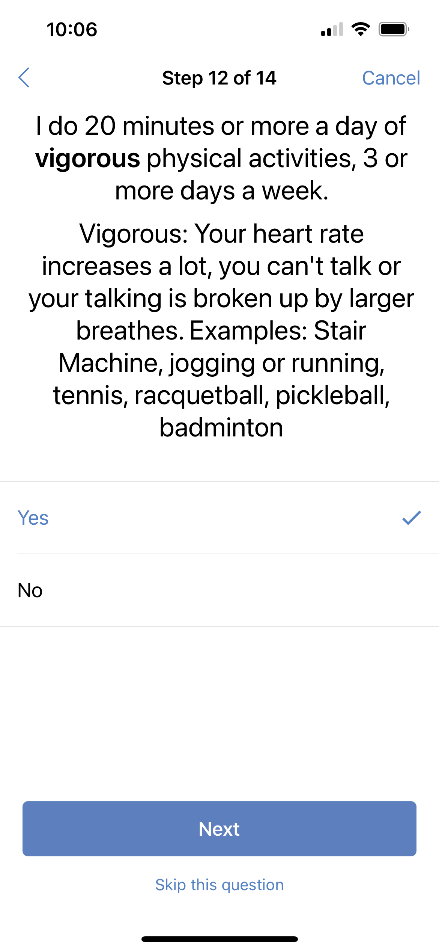

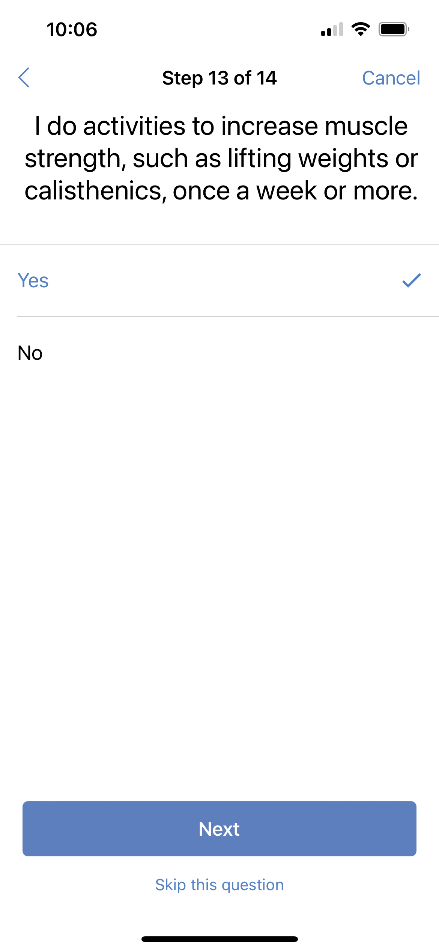

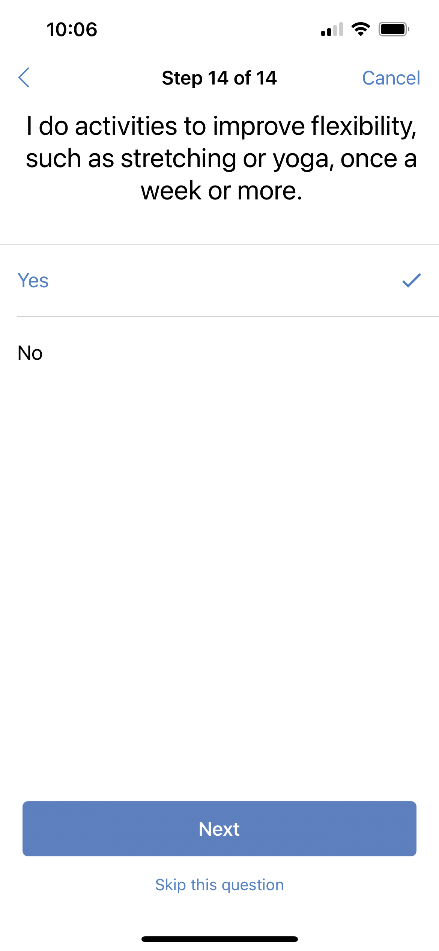

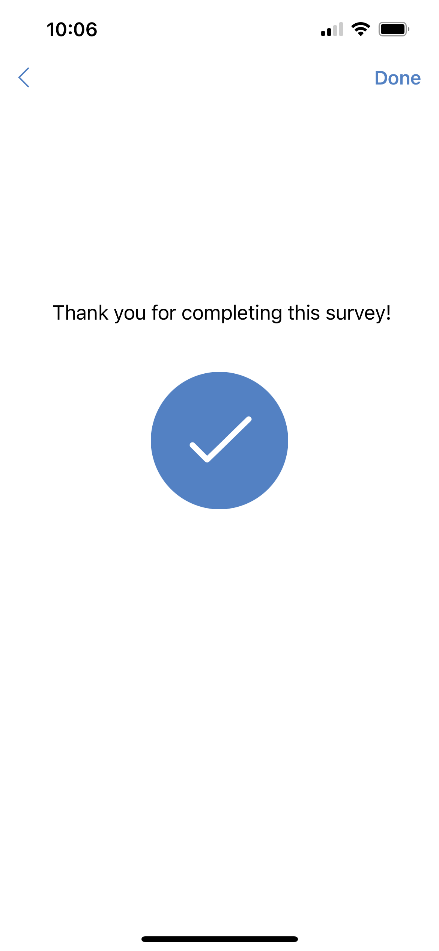
**

**Falls and Hospitalization Survey:**

**
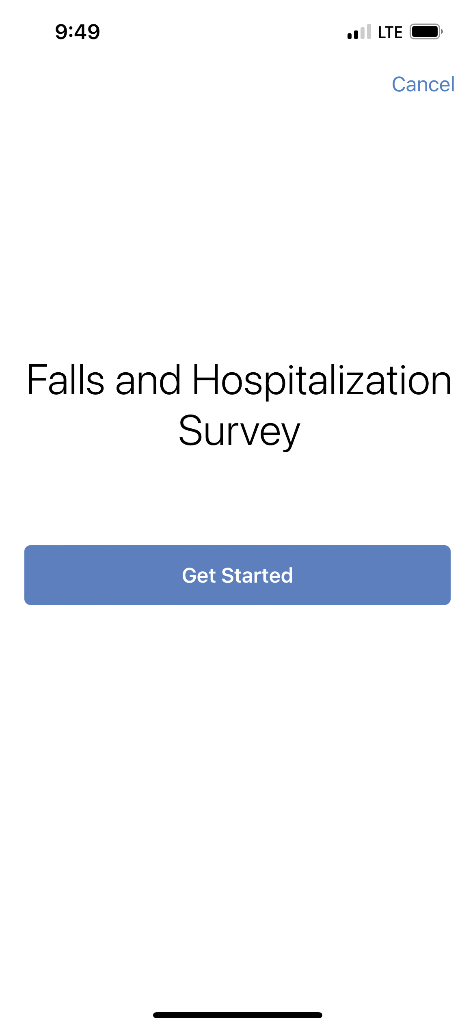
**

**
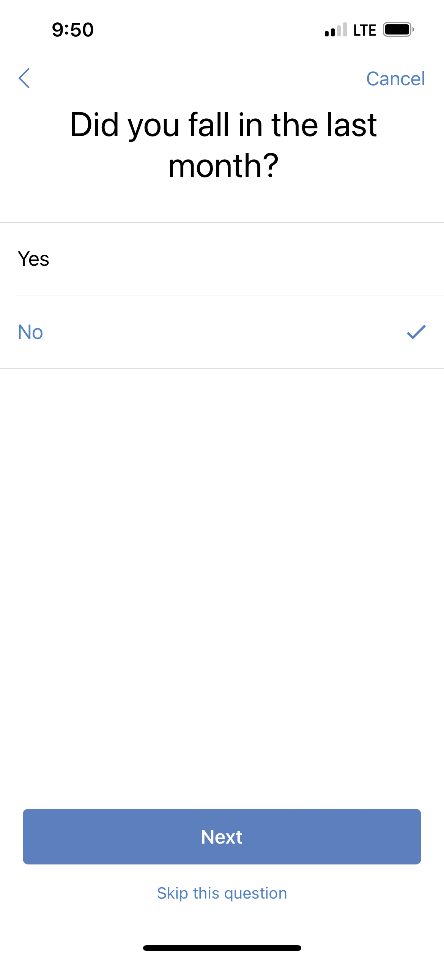

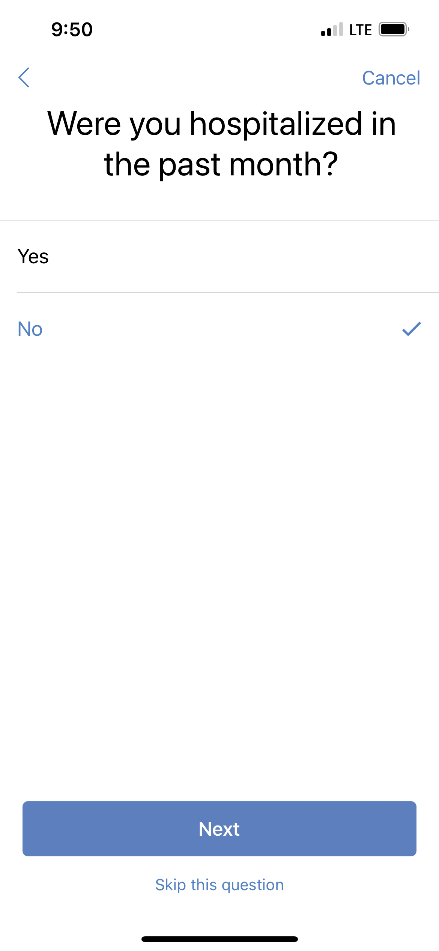

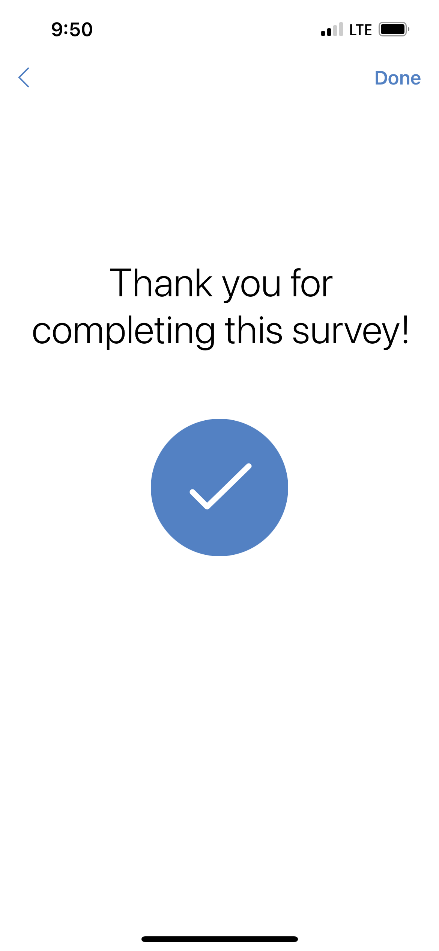
**

**
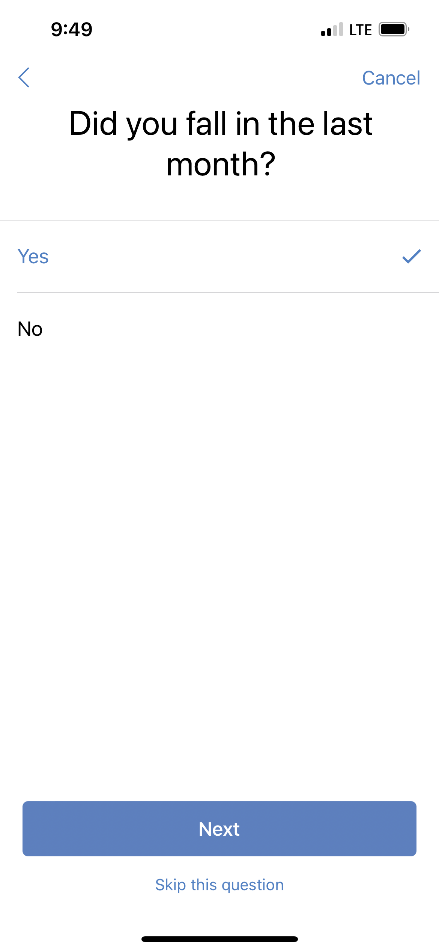

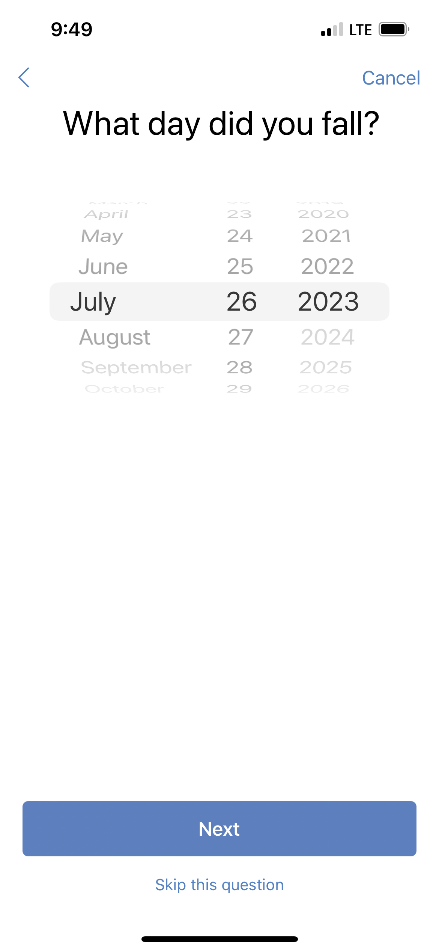

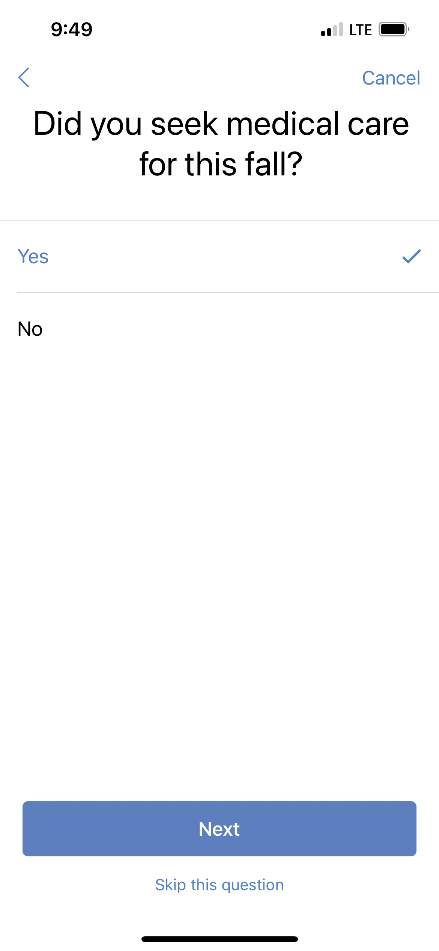
**

**
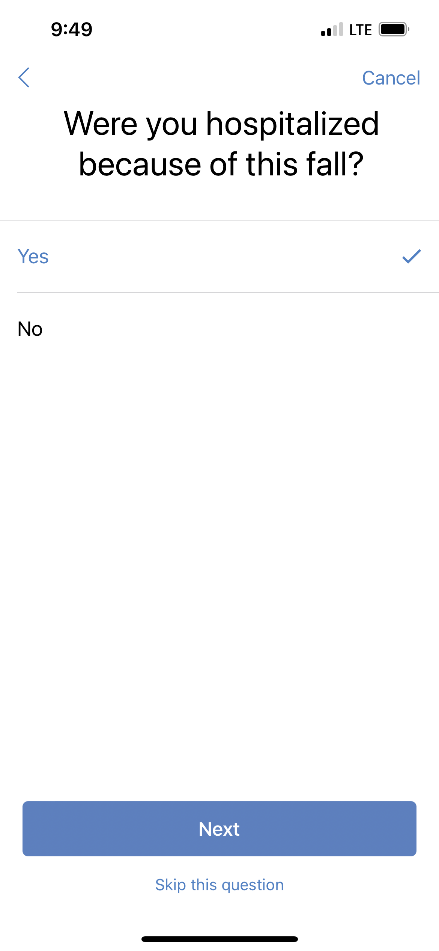

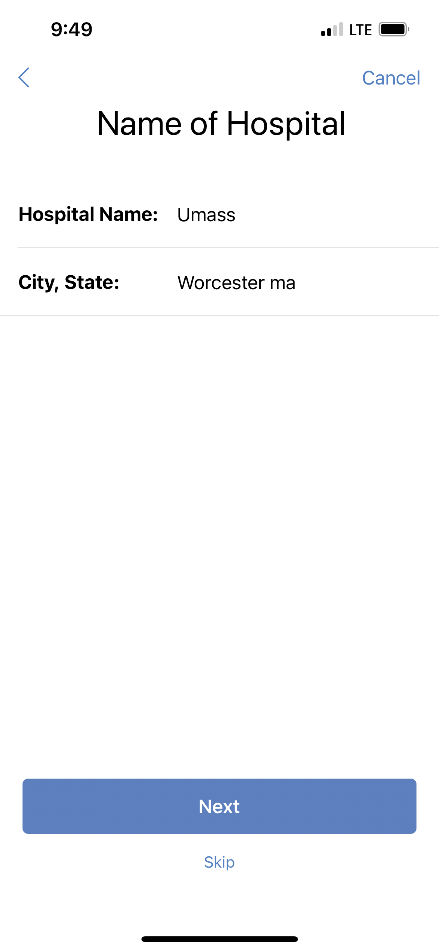

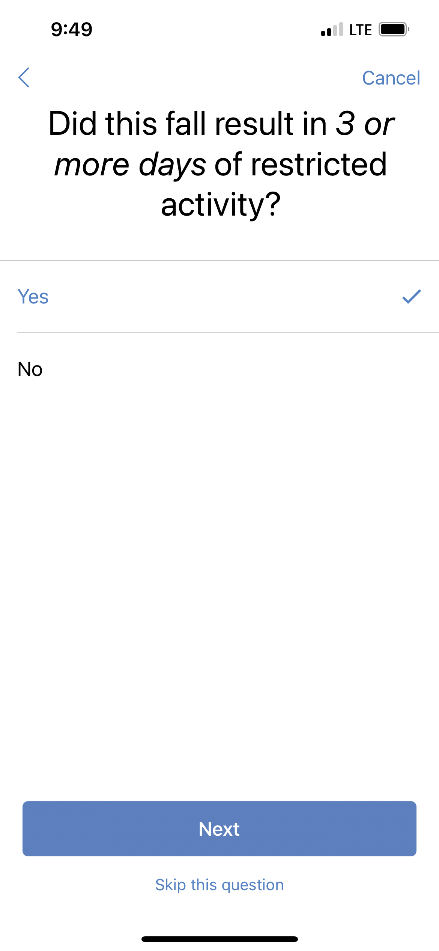
**

**
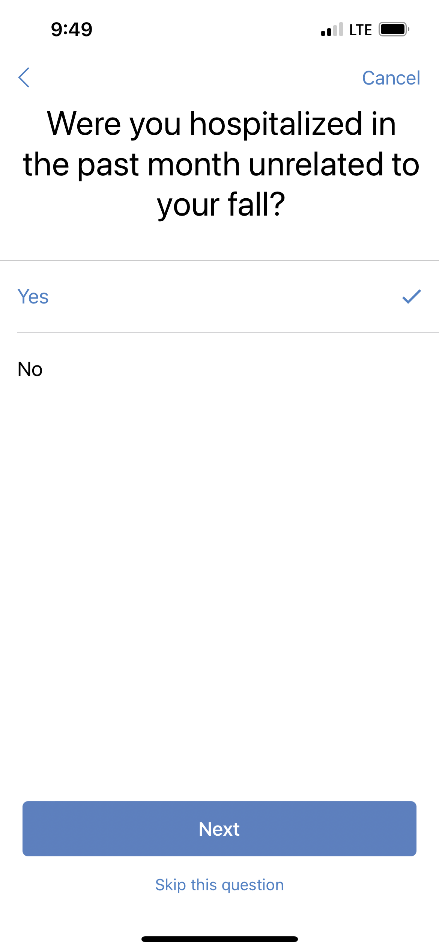

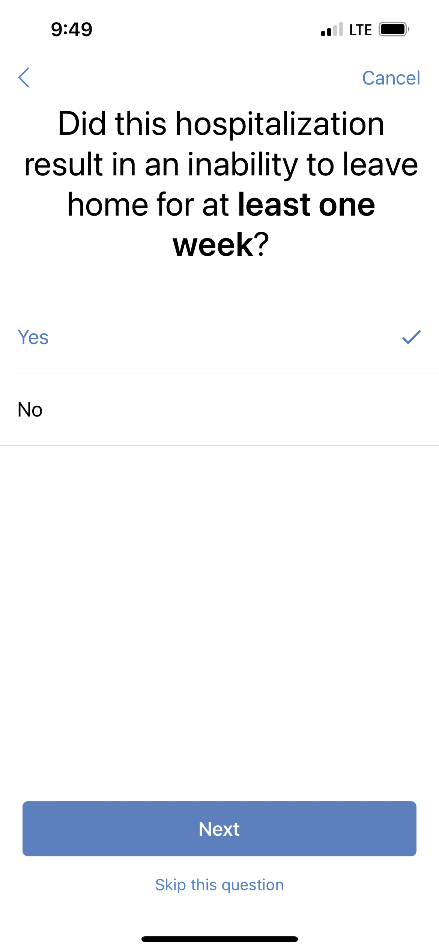

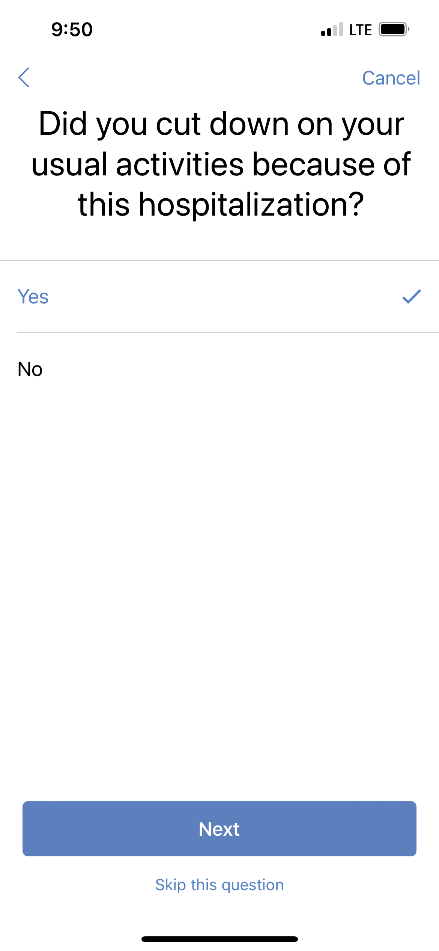
**

**
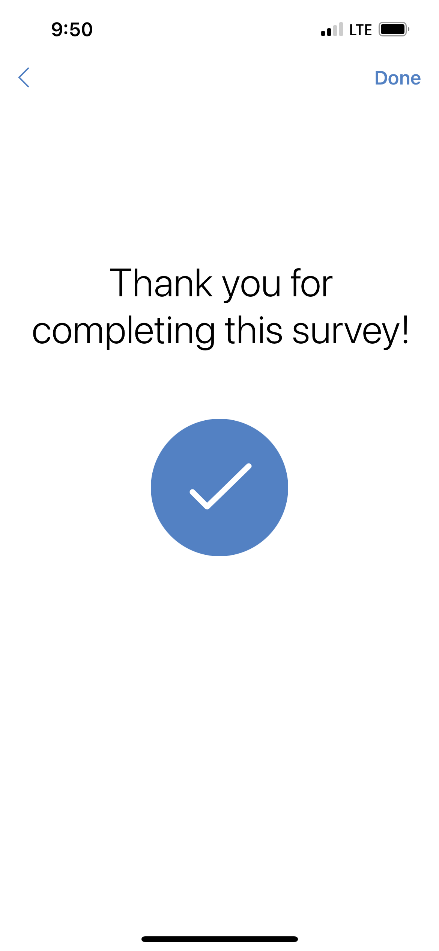
**

**Cognitive Functions Survey:**

**Cognition Abilities Survey:**

**Trail Making Test:**

**STROOP:**

**Two Finger Tap:**

**Gait:**

**Loneliness Survey:**

**Social Support Survey 2:**

**Resilience Survey:**

**Social Support Survey 1:**
