# Supplementary material for: Smartphone App–Based Survey Deployment Patterns and Longitudinal Response Rate: Randomized Controlled Trial
Source: J Med Internet Res. 2025 Oct 10;27:e73972. doi: 10.2196/73972 (PMC12552817; doi:10.2196/73972)
Supplement: Multimedia Appendix 5 [file jmir_v27i1e73972_app5.docx]

**Figure S1.** Correlation between primary and secondary outcomes.


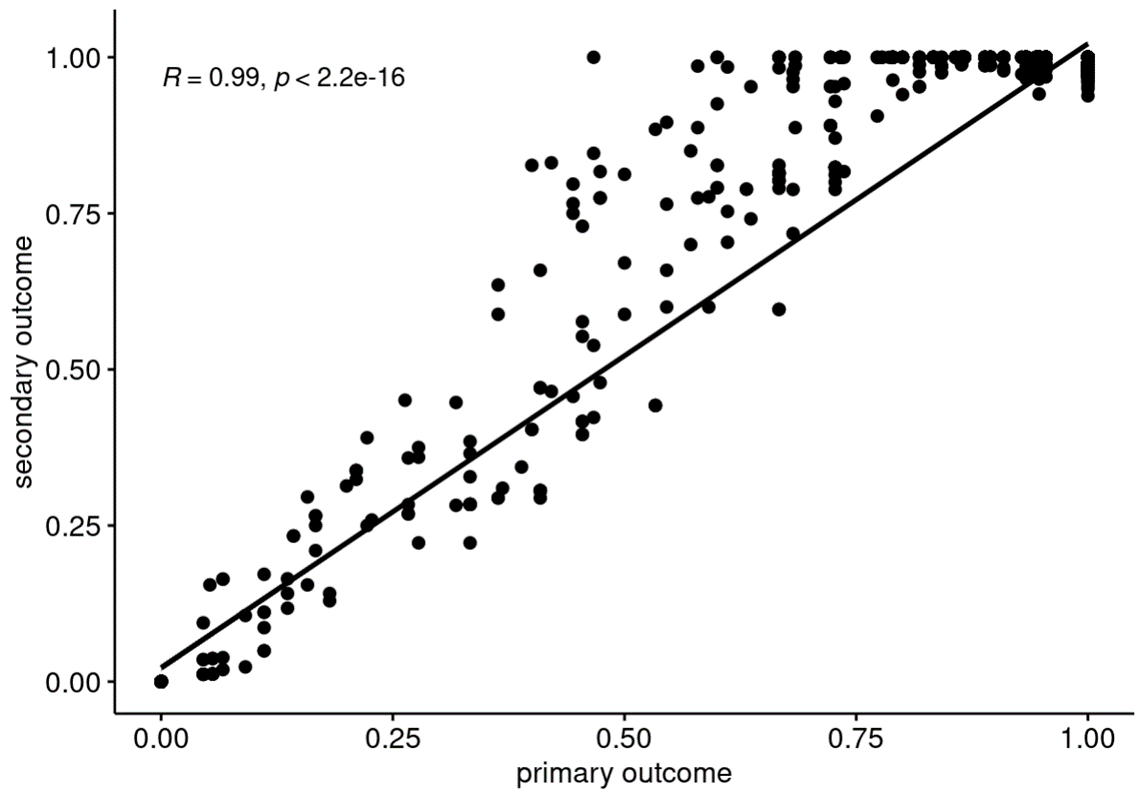


Primary outcome was defined as the proportion of surveys returned per participant in each time period. Secondary outcome was defined as the proportion of questions/tasks completed per participant in each time period.

**Figure S2.** Mean of drop-out rate by randomization groups across 4 time periods.


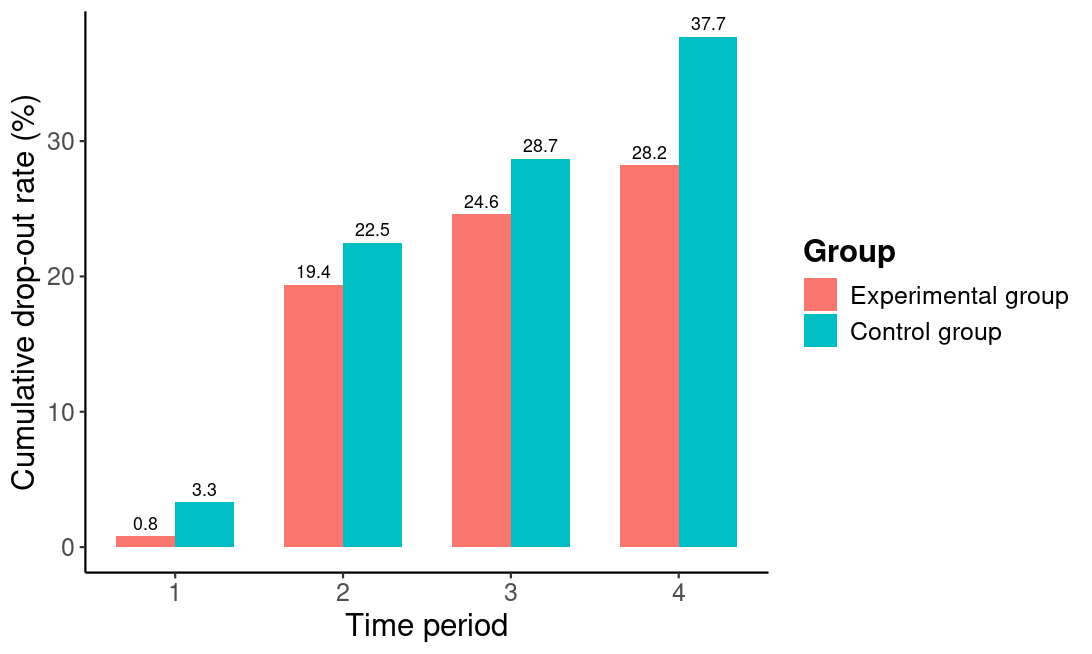


Time period: 1=week 0-8; 2=week 8-16; 3=week 16-24; 4=week 24-32.

Drop-out was defined as a participant not returning any surveys within a time period.

**Table S1.** Characteristics of eFHS participants not in the trial compared with trial participants.

| **Characteristics** | **Other eFHS participants (n=128)** | **Trial participants (n=492)** | ***P*** |
| --- | --- | --- | --- |
| Age, years, mean (SD) | 74.3 (5.1) | 73.9 (6.3) | .59 |
| Women, n (%) | 68 (53.1) | 284 (57.7) | .40 |
| Race/ethnicity, n (%)  Non-Hispanic White  Black  Asian  Hispanic | 118 (92.2)  4 (3.1)  5 (3.9)  1 (0.8) | 414 (84.1)  30 (6.1)  35 (7.1)  13 (2.6) | .13 |
| Bachelor’s degree or higher, n (%) | 76 (59.4) | 327 (66.5) | .16 |
| Marital status, married, n (%) | 84 (65.6) | 340 (69.4) | .48 |
| Participating as couple, n (%) | 12 (24.0) | 98 (19.9) | .62 |
| Phone type, iPhone, n (%) | 96 (75.0) | 382 (77.6) | .88 |
| Smartwatch user, n (%) | 68 (53.1) | 287 (60.2) | .18 |
| Retired, n (%) | 95 (74.2) | 337 (68.6) | .26 |
| Annual income, n (%)  <$35,000  $35,000-$74,999  ≥$75,000 | 9 (8.4)  29 (27.1)  69 (64.5) | 42 (10.7)  110 (28.1)  239 (61.1) | .73 |
| Subjective health, very good or excellent, n (%) | 99 (77.3) | 337 (68.6) | .07 |

**Table S2**. Effect of survey deployment patterns on the binary outcome* for survey response rate.

| **Time period**^†^ | **Odds ratio (95% CI)^#^** | ***P* of group-by-time interaction** |
| --- | --- | --- |
| 1 | 1.00 (0.93, 1.07) | <.001 |
| 2 | 1.02 (0.96, 1.09) |  |
| 3 | 1.05 (0.99, 1.12) |  |
| 4 | 1.08 (1.01, 1.15) |  |

*Each survey represented a binary outcome: Returned surveys were coded as “1”, non-returned as “0”.

^†^Time period: 1=week 0-8; 2=week 8-16; 3=week 16-24; 4=week 24-32.

**^#^**Odds ratios were calculated as odds of survey response in experimental group divided by odds of survey response in control group.

**Table S3.** Comparison of primary and secondary outcomes stratified by age-group.

| **Age>75 years (n=204)** | | | | |
| --- | --- | --- | --- | --- |
| **Time period**^†^ | **Experimental group (n=104)** | **Control group (n=100)** | **Difference in proportions^#^** | ***P* of group-by-time interaction** |
| **Primary outcome (%)**^$^**: proportion of surveys returned** (**95% CI**) | | | | |
| 1 | 65.4 (56.7, 74.1) | 71.6 (62.6, 80.5) | -6.2 (-17.4, 5.0) | .06 |
| 2 | 58.8 (50.6, 67.1) | 62.5 (53.9, 71.0) | -3.6 (-14.1, 6.8) |  |
| 3 | 52.3 (44.0, 60.5) | 53.3 (44.8, 61.8) | -1.0 (-11.5, 9.4) |  |
| 4 | 45.7 (37.0, 54.4) | 44.2 (35.2, 53.2) | 1.5 (-9.6, 12.7) |  |
| **Secondary outcome (%)**^$^: **proportion of questions/tasks completed** (**95% CI**) | | | | |
| 1 | 66.4 (57.7, 75.1) | 74.2 (65.3, 83.2) | -7.8 (-19.1, 3.4) | .02 |
| 2 | 60.1 (51.9, 68.3) | 64.6 (56.2, 73.1) | -4.5 (-15.0, 5.9) |  |
| 3 | 53.8 (45.6, 62.0) | 55.0 (46.5, 63.5) | -1.2 (-11.6, 9.2) |  |
| 4 | 47.5 (38.8, 56.2) | 45.4 (36.4, 54.4) | 2.1 (-9.1, 13.3) |  |
| **Age≤75 years (n=288)** | | | | |
| **Time period**^†^ | **Experimental group (n=144)** | **Control group (n=144)** | **Difference in proportions^#^** | ***P* of group-by-time interaction** |
| **Primary outcome (%)**^$^**: proportion of surveys returned** (**95% CI**) | | | | |
| 1 | 83.3 (76.7, 89.9) | 79.2 (72.5, 85.9) | 4.1 (-4.5, 12.7) | .02 |
| 2 | 77.8 (71.6, 84.1) | 71.2 (64.9, 77.6) | 6.6 (-1.4, 14.6) |  |
| 3 | 72.3 (66.1, 78.6) | 63.3 (57.0, 69.6) | 9.1 (1.0, 17.1) |  |
| 4 | 66.8 (60.2, 73.4) | 55.3 (48.6, 62.0) | 11.5 (2.9, 20.1) |  |
| **Secondary outcome (%)**^$^: **proportion of questions/tasks completed** (**95% CI**) | | | | |
| 1 | 85.2 (78.6, 91.9) | 80.9 (74.2, 87.6) | 4.3 (-4.3, 12.9) | .04 |
| 2 | 79.7 (73.4, 85.9) | 73.1 (66.8, 79.4) | 6.6 (-1.4, 14.6) |  |
| 3 | 74.1 (67.9, 80.4) | 65.3 (59.0, 71.6) | 8.8 (0.9, 16.8) |  |
| 4 | 68.6 (61.9, 75.2) | 57.4 (50.7, 64.1) | 11.1 (2.5, 19.8) |  |

^†^Time period: 1=week 0-8; 2=week 8-16; 3=week 16-24; 4=week 24-32.

^$^Estimated outcomes were derived from a linear mixed-effects model analyzing repeated measurements of primary/secondary outcomes across four time points per participant. The model included fixed effects for randomization group, timepoint (time period 1-4), phone type (iPhone vs. Android), and a randomization group-by-time interaction. Random intercepts were included for each couple and individual to account for correlation within couples and repeated measures of individuals.

^#^Differences estimated as outcomes of experimental group minus outcomes of control group.

**Table S4.** Comparison of primary and secondary outcomes stratified by sex.

| **Women (n=284)** | | | | |
| --- | --- | --- | --- | --- |
| **Time period**^†^ | **Experimental group (n=148)** | **Control group (n=136)** | **Difference in proportions^#^** | ***P* of group-by-time interaction** |
| **Primary outcome (%)**^$^**: proportion of surveys returned** (**95% CI**) | | | | |
| 1 | 76.6 (69.3, 83.8) | 78.7 (70.9, 86.4) | -2.1 (-11.5, 7.4) | .01 |
| 2 | 70.9 (64.1, 77.7) | 70.1 (62.8, 77.5) | 0.8 (-8.0, 9.6) |  |
| 3 | 65.2 (58.4, 72.1) | 61.6 (54.3, 69.0) | 3.6 (-5.2, 12.4) |  |
| 4 | 59.5 (52.3, 66.8) | 53.1 (45.4, 60.9) | 6.4 (-3.0, 15.9) |  |
| **Secondary outcome (%)**^$^: **proportion of questions/tasks completed** (**95% CI**) | | | | |
| 1 | 78.5 (71.2, 85.7) | 80.6 (72.9, 88.4) | -2.2 (-11.7, 7.3) | .01 |
| 2 | 72.8 (65.9, 79.6) | 72.1 (64.7, 79.4) | 0.7 (-8.1, 9.5) |  |
| 3 | 67.1 (60.2, 73.9) | 63.5 (56.2, 70.8) | 3.6 (-5.2, 12.3) |  |
| 4 | 61.4 (54.1, 68.6) | 54.9 (47.1, 62.7) | 6.4 (-3.0, 15.9) |  |
| **Men (n=208)** | | | | |
| **Time period**^†^ | **Experimental group (n=100)** | **Control group (n=108)** | **Difference in proportions^#^** | ***P* of group-by-time interaction** |
| **Primary outcome (%)**^$^**: proportion of surveys returned** (**95% CI**) | | | | |
| 1 | 74.3 (64.5, 84.1) | 72.6 (63.2, 82.0) | 1.7 (-10.9, 14.3) | .12 |
| 2 | 68.0 (58.7, 77.3) | 64.2 (55.3, 73.1) | 3.8 (-8.1, 15.6) |  |
| 3 | 61.7 (52.4, 71.0) | 55.9 (46.9, 64.8) | 5.8 (-6.0, 17.6) |  |
| 4 | 55.3 (45.5, 65.2) | 47.5 (38.1, 56.9) | 7.8 (-4.8, 20.5) |  |
| **Secondary outcome (%)**^$^: **proportion of questions/tasks completed** (**95% CI**) | | | | |
| 1 | 75.3 (65.3, 85.2) | 75.0 (65.5, 84.5) | 0.2 (-12.5, 13.0) | .08 |
| 2 | 69.2 (59.8, 78.5) | 66.5 (57.5, 75.4) | 2.7 (-9.2, 14.5) |  |
| 3 | 63.1 (53.7, 72.4) | 57.9 (49.0, 66.9) | 5.1 (-6.7, 17.0) |  |
| 4 | 56.9 (47.0, 66.9) | 49.4 (39.9, 58.9) | 7.6 (-5.2, 20.3) |  |

^†^Time period: 1=week 0-8; 2=week 8-16; 3=week 16-24; 4=week 24-32.

^$^Estimated outcomes were derived from a linear mixed-effects model analyzing repeated measurements of primary/secondary outcomes across four time points per participant. The model included fixed effects for randomization group, timepoint (time period 1-4), age-group (≤75 years vs. >75 years), phone type (iPhone vs. Android), and a randomization group-by-time interaction. Random intercepts were included for each couple and individual to account for correlation within couples and repeated measures of individuals.

^#^Differences estimated as outcomes of experimental group minus outcomes of control group.
